# Cerebellar Transcranial Alternating Current Stimulation in the Theta Band Prevents Recall of the Initial Fear Association After Extinction Training

**DOI:** 10.1002/hbm.70556

**Published:** 2026-06-03

**Authors:** Giorgi Batsikadze, Zsofia Spisak, Philippe Zeidan, Michael Klein, Enzo Nio, Thomas M. Ernst, Nicolas Diekmann, Sophia Göricke, Sen Cheng, Christian J. Merz, Fatemeh Yavari, Michael A. Nitsche, Andreas Thieme, Dagmar Timmann

**Affiliations:** ^1^ Department of Neurology and Center for Translational Neuro‐ and Behavioral Sciences (C‐TNBS) University Hospital Essen Essen Germany; ^2^ Institute for Neural Computation Faculty of Computer Science, Ruhr University Bochum Bochum Germany; ^3^ Institute of Diagnostic and Interventional Radiology and Neuroradiology, Essen University Hospital Essen Germany; ^4^ Ruhr University Bochum, Faculty of Psychology, Institute of Cognitive Neuroscience Department of Cognitive Psychology Bochum Germany; ^5^ Department of Psychology and Neurosciences Leibniz Research Centre for Working Environment and Human Factors Dortmund Germany; ^6^ German Center for Mental Health (DZPG) Center Bochum Germany; ^7^ Bielefeld University, University Hospital OWL, Protestant Hospital of Bethel Foundation University Clinic of Psychiatry and Psychotherapy Bielefeld Germany

**Keywords:** associative learning, aversive conditioning, fear conditioning, fear prediction error, fMRI, non‐invasive brain stimulation, transcranial alternating current stimulation

## Abstract

Although the neural network underlying fear extinction has been extensively studied, the cerebellum's role has received little attention – despite its well‐established involvement in associative learning. Our study therefore aimed to provide additional evidence that the cerebellum is part of the circuitry supporting fear‐extinction processes, and to get a better understanding of how the cerebellum may contribute to fear extinction learning. In this study, 6 Hz cerebellar transcranial alternating current stimulation (ctACS) or sham stimulation was applied during extinction training in a two‐day differential fear conditioning paradigm in young, healthy participants undergoing 3T fMRI, using a double‐blind randomized design. Acquisition and extinction training occurred on day 1, followed by extinction recall on day 2. Skin conductance responses showed that 6 Hz ctACS applied during extinction training reduced spontaneous fear recovery during recall. During extinction training, differential fMRI activation (CS+ > CS‐) was significantly higher in the occipital cortex in the verum compared to the sham group. During recall, differential fMRI activation was significantly higher in the precentral gyrus in the sham compared to the verum group at the time the aversive unconditioned response (US) was expected but did not occur. Furthermore, in recall, parametric modulation based on trial‐by‐trial model‐derived prediction errors for no‐US events revealed significantly higher activation in frontal cortical areas, including the anterior cingulate cortex, and parietal cortical areas in the sham compared to the verum group. Volume of interest analyses showed significantly higher beta values towards the CS+ compared to the CS‐ in the sham group, but not in the verum group in the right insula related to the prediction of the US and its unexpected omission in early recall. Although direct stimulation effects cannot be ruled out, 6 Hz ctACS‐related increases in activation in visual regions during extinction training may indicate enhanced attention to CS‐related visual and/or contextual cues. Furthermore, 6 Hz ctACS facilitated the downregulation of brain regions involved in fear conditioning during recall, potentially reducing spontaneous recovery. Future studies are warranted to further evaluate whether enhancement of cerebellar theta oscillations can help to stabilize extinction effects and therefore support exposure therapy.

## Key Points

- 6 Hz cerebellar tACS during extinction training reduced spontaneous fear recovery during recall.
- 6 Hz cerebellar tACS decreased activation within the fear extinction network during recall.
- Targeting cerebellar theta oscillations may stabilize extinction effects.

## Graphical abstract

In this 3 T fMRI study, 6 Hz cerebellar transcranial alternating current stimulation (ctACS) was applied to modulate cerebellar function during extinction training in healthy participants. ctACS applied during extinction training reduced spontaneous fear recovery during recall. fMRI analyses revealed increased activation in occipital regions during extinction training and decreased activation in parts of the fear acquisition network during recall. Future studies are warranted to investigate whether targeting cerebellar theta oscillations may stabilize extinction memory.

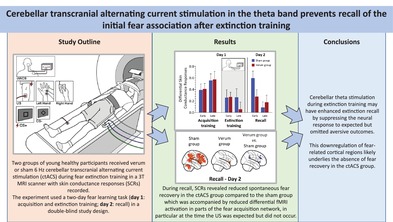

## 1 Introduction

The cerebellum is well known for its contribution to associative learning. The best‐known example is eyeblink conditioning, a form of motor learning (De Zeeuw and Ten Brinke 2015). The cerebellum is also involved in associative learning in the cognitive and emotional domains (Diedrichsen et al. 2019; Guell and Schmahmann 2020; Schmahmann 2019). One important emotion is fear. The cerebellum has been shown to contribute to learning of new fear associations (Hwang et al. 2022; Urrutia Desmaison et al. 2023). However, despite extended research on the neural network involved in extinction of learned fear, the likely contribution of the cerebellum to fear extinction has been examined only to a limited extent (Doubliez et al. 2023). Fear extinction is of general interest, because deficient fear extinction is thought to contribute to a variety of anxiety disorders including posttraumatic stress disorder and others, and is also at the core of exposure therapy frequently used to treat anxiety disorders (Sep et al. 2023; VanElzakker et al. 2014).

The cerebellum is well suited to support extinction learning due to its anatomical connections with key regions of the fear extinction network, including the ventromedial prefrontal cortex (vmPFC), hippocampus, insular cortex, striatum, amygdala, and midbrain periaqueductal gray (Boll et al. 2013; J. Li et al. 2011; S. S. Li and McNally 2014). Recent work in rodents shows that the cerebellum regulates fear extinction via monosynaptic projections from the fastigial nuclei to the periaqueductal gray (Frontera et al. 2020; Lawrenson et al. 2022), but also through thalamo‐prefrontal interactions (Frontera et al. 2023). In human functional brain imaging studies (fMRI) activations of the cerebellum have been observed related to fear extinction learning (Batsikadze et al. 2022; Chang et al. 2015; Nio et al. 2025). There is also initial evidence that extinction learning is reduced in patients with cerebellar disease (Batsikadze et al. 2024; Maschke et al. 2002).

The first aim of our study was to provide further experimental evidence that the cerebellum has to be included as part of the neural circuitry underlying extinction of conditioned fear responses in humans. The second aim was to get a better understanding of how the cerebellum may contribute. To achieve this aim, cerebellar transcranial alternating current stimulation (cerebellar tACS, ctACS) was used to modulate cerebellar function during extinction learning in healthy human participants in an MR scanner (Wessel et al. 2023). tACS, when administered at frequencies matching natural neuronal oscillations, can synchronize (“entrain”) intrinsic oscillations and enhance functional connectivity (Antal and Herrmann 2016; Bachinger et al. 2017). Animal studies show that this is equally the case for the cerebellum (Asan et al. 2020; Kang et al. 2023).

We targeted cerebellar theta oscillations with 6 Hz ctACS, aligning with the response frequency of cerebellar granule and Golgi cells (D'Angelo et al. 2013; Gandolfi et al. 2013). Theta activity is well known to contribute to visuomotor adaptation and associative learning in humans and animals (Jonker et al. 2021; Tzvi et al. 2022). More specifically, a correlation between spontaneous cerebellar theta activity in the 4–7 Hz frequency range and successful extinction of conditioned eyeblink responses has been found in guinea pigs (Y. J. Wang et al. 2014). Furthermore, a decrease in cerebellar theta activity following CS exposure has been linked to the spontaneous reappearance of previously extinguished eyeblink responses (H. Wang et al. 2019).

We found that theta ctACS during extinction training strengthened extinction recall, which was accompanied by significant changes of cerebral activations during extinction learning and recall. These findings provide further support that the cerebellum is involved in fear extinction learning and modulates cerebral areas involved in fear extinction. The cerebellum is increasingly used as a target for invasive and non‐invasive brain stimulation. Findings may therefore also enable new treatment possibilities for anxiety disorders.

## 2 Materials and Methods

### 2.1 Subjects

A total of 55 young, healthy, right‐handed, non‐smoking participants (29 men/26 women, 23.5 ± 3.5 years) were recruited to participate in the experiment. Sixteen participants were excluded from the initial group: six participants had to be excluded due to technical errors (volume orientation mix‐ups or loss of adjustment volume settings); six participants due to moderate or higher depression, anxiety or stress scores based on the Depression Anxiety Stress Scale‐21 (DASS‐21) questionnaire (Lovibond and Lovibond 1995); one participant was excluded for not attending the experiment on the second day; one was excluded due to an incidental finding (cavernoma in the temporal lobe); one for taking oral contraceptives, which were not allowed as part of the study protocol, and one for insufficient blinding based on results of the stimulation side‐effect questionnaire (reported scores exceeded three standard deviations above the mean on 5 of the 6 skin‐sensation‐related items).

39 participants (21 men/18 women, 23.5 ± 3.6 years) were included in the final skin conductance response (SCR) data analysis. Four of the 39 participants were excluded from the fMRI analysis because excessive motion‐related artifacts were detected during data inspection. Thus, 35 participants (19 men/16 women, 23.7 ± 3.2 years) were included in the final fMRI data analysis. DASS‐21 scores of the participants included in the final analysis fell within the normal‐to‐mild range: depression, median 2 (IQR [interquartile range] 0–4, range 0–10), anxiety, median 2 (IQR 0–4, range 0–8), stress, median 6 (IQR 2–10, range 0–12).

Each participant underwent a neurological examination prior to the start of the experiment, and their depression, anxiety, and stress levels were assessed using the DASS‐21 questionnaire (Henry and Crawford 2005; Norton 2007). Participants who had a history of neurological disease, metallic head implants, were pregnant, or had taken central nervous system‐active medication were excluded from the study. Women taking oral contraceptives were also excluded from the study to avoid any effects on fear‐conditioning processes caused by changes in circulating sex hormones (Merz et al. 2018; Velasco et al. 2019). All participants were right‐handed based on the Edinburgh handedness inventory (Oldfield 1971), naïve to both brain stimulation and fear learning procedures, and were instructed to refrain from alcohol for at least 24 h before the experiment and for its duration.

The study was approved by the Ethics Committee of the University Hospital Essen (proposal ID 16–7255‐BO) and was in line with the principles laid down in the Declaration of Helsinki. We obtained informed consent from all participants, and they were compensated with 80 Euro for their participation.

### 2.2 Experimental Procedures

**FIGURE 1:**
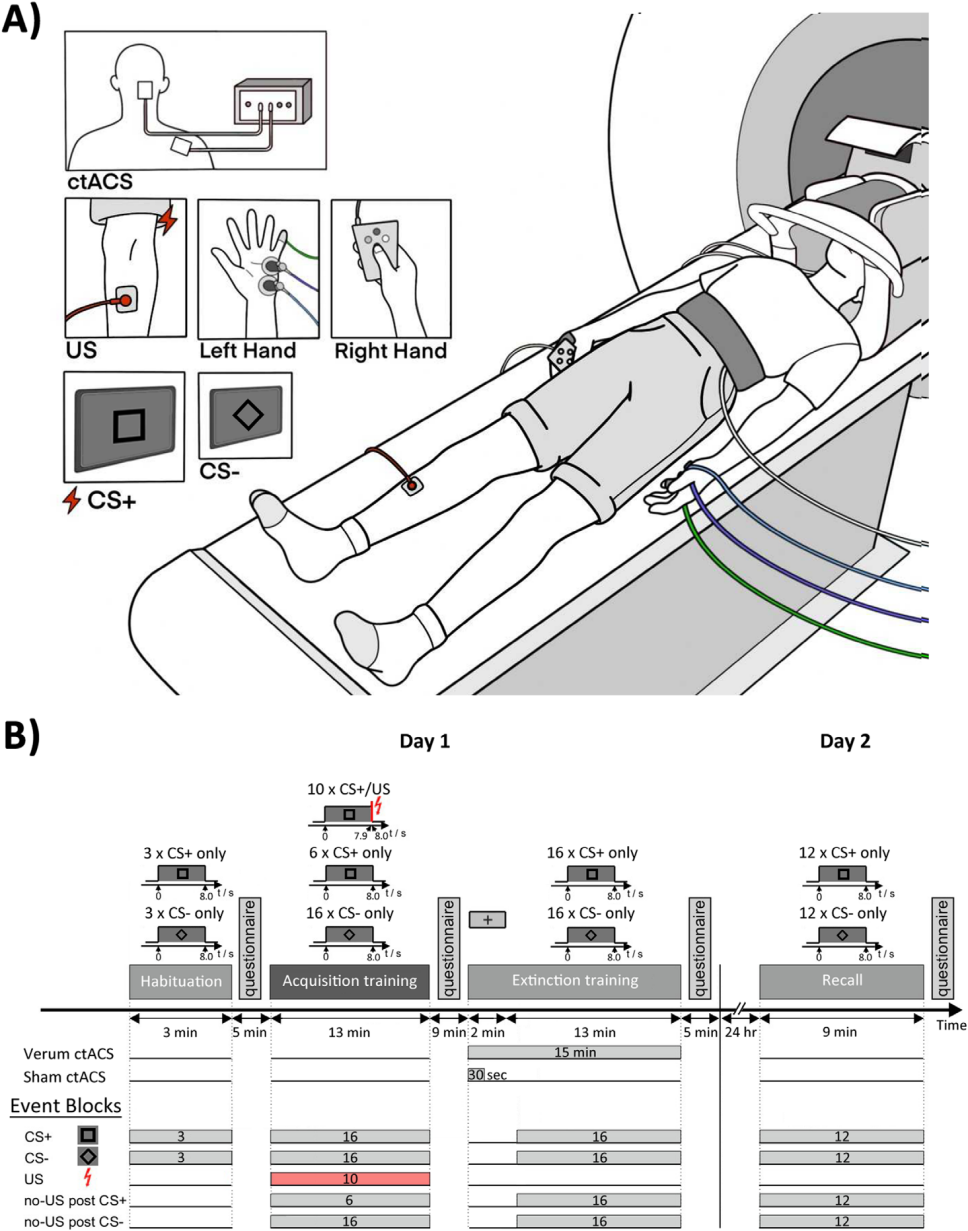
(A) Experimental paradigm and (B) event blocking scheme. During the experiment, two different geometrical figures, a diamond and a square, were presented as conditioned stimuli (CS), while an electric shock was used as an unconditioned stimulus (US). A black cross image (“fixation cross”) was displayed against a neutral gray background during the first 2 min prior to the first CS picture onset in the extinction phase. Additional information regarding the experiment can be found in the text.

The differential fear conditioning paradigm used in this study was based on the publication by Ernst et al. (2019). Differential fear conditioning was performed using two CS: two pictures of black geometric figures (a square and a diamond shape) of identical brightness (Figure 1A) on a grey background. The CS+ was followed by an aversive US (paired CS+/US trial) during fear acquisition training in 62.5% of the trials. The CS‐ was never followed by the US. The use of the two CS figures was pseudorandomly counterbalanced across participants. The experiment was performed on two consecutive days (Figure 1B). Day 1 consisted of three phases: “habituation” (3 CS+ only trials, 3 CS‐ only trials), “acquisition training” (10 paired CS+/US trials, 6 CS+ only trials, 16 CS‐ only trials), and “extinction training” (16 CS+ only trials, 16 CS‐ only trials). Day 2 consisted of the recall phase (12 CS+ only trials, 12 CS‐ only trials) and took place about 24 h after the start of day 1 to maintain a consistent interval between sessions. The presentation order of trial types in each phase was pseudorandomized, with two restrictions: firstly, the first two trials and the last trial in the acquisition training were paired CS+/US trials, and secondly, the number of events of each kind was kept equal in the first and second halves of each phase. The order of events was identical for all participants during habituation, acquisition, and extinction training. During recall, the initial event was counterbalanced between CS+ and CS‐ trials.

The electric shock was generated by a constant current stimulator (DS7A, Digitimer Ltd., London, UK) and applied to the right shin via a concentric (ring‐shaped) bipolar surface electrode with 6 mm conductive diameter and a central platinum pin (WASP electrode, Specialty Developments, Bexley, UK). The electrode position was marked with a permanent marker on day 1 to use the same electrode position on day 2. The 100 ms US consisted of a short train of four consecutive 500 μs current pulses (maximum output voltage: 400 V) with an interpulse interval of 33 ms. Before the start of MRI measurements, the US intensity for each participant was determined. The stimulation intensity was increased gradually, and participants were asked to rate the perceived sensation on a nine‐point Likert scale ranging from “not unpleasant” to “very unpleasant” until a score of 8 out of 9 (i.e., “unpleasant but not painful”) was reached. To avoid a reduction in the conditioned responses caused by habituation to the US, the individual thresholds were increased by 20% (Batsikadze et al. 2022; Inoue et al. 2020). The average US intensity was 5.35 ± 6.03 mA (range 1 mA—34.8 mA), and there were no significant differences in the applied current between the two groups (independent samples *t*‐test: *t*_(37)_ = 0.711, *p* = 0.241).

Prior to each phase, the participants were provided with on‐screen instructions, notifying them of the upcoming visual stimuli. Prior to habituation, they were informed that electric shocks would not be administered. Prior to the acquisition training, they were informed that electric shocks would be administered during this phase, and that if they detected a pattern between stimuli, it would remain consistent throughout the experiment. On the second day, participants were again reminded that any pattern perceived during day 1 would remain the same on day 2. All participants had to confirm that they read and comprehended the instructions.

Each trial consisted of an 8 s CS presentation. In the case of reinforced trials, a 100 ms aversive US was presented after 7.9 s and coterminated with the CS. A black cross image (“fixation cross”) was displayed against a neutral gray background before the first CS picture onset and during the first two minutes of ctACS prior to the extinction phase. Intertrial intervals were randomized between 14.3 s and 17.9 s. Each experimental phase was performed within a separate session of fMRI data acquisition.

Participants had to answer a questionnaire following each phase of the experiment. As outlined in more detail in Batsikadze et al. (2022), they were asked to rate the hedonic valence, emotional arousal, fear, and expectancy of an US on a nine‐step Likert scale. The scale ranged from “*very pleasant*” to “*very unpleasant*,” “*very calm*” to “*very nervous*,” “*not afraid*” to “*very afraid*,” and “*US not expected*” to “*US expected*” respectively. After undergoing fear acquisition training, participants were asked to rate the unpleasantness of the US on a Likert scale ranging from 1 (“not unpleasant”) to 9 (“very unpleasant”) and to estimate the mean probability (in %) that an US occurred following the presentation of the CS (CS/US contingency).

Additionally, before and after the ctACS stimulation session, participants had to respond to questions about possible stimulation side effects (headache, neck pain, back pain, blurred vision, scalp irritation, scalp tingling, scalp itching, accelerated heartbeat, burning sensation, hot flashes, vertigo, sudden mood change, fatigue, phosphenes) and rate them on a Likert scale from 1 (“absent”) to 9 (“strong”) (adapted from Brunoni et al. 2011). Participants used an MRI‐compatible button box with their right hand to give answers to the questions that were projected onto the screen located inside the MRI scanner.

### 2.3 Physiological Data Acquisition

Throughout the experiment, SCRs, pulse, and breathing rate were acquired using MRI‐compatible skin conductance, pulse oximetry, and differential air pressure modules (MP160, BIOPAC Systems Inc., Goleta, CA). The sampling rate was set at 2 kHz. Two skin conductance electrodes were attached to the participant's left hypothenar, approximately 20 mm apart. The pulse oximetry sensor was clipped to the participant's left index finger. A respiratory belt was attached to the participant's lower abdomen using a hook‐and‐loop belt.

### 2.4 Modeling of Prediction and Prediction Error Values

An artificial agent was trained to predict the likelihood of a shock for a given visual input in a virtual version of the experiment as described in Batsikadze et al. (2022) and summarized in the following. The model was based on reinforcement learning (Sutton and Barto 2018) and consisted of a deep neural network. The model hyper‐parameters were fit to SCRs recorded in the experiment, which served as a read‐out of the participants' expectation of an US. The resulting model was used to derive predictions for the likelihood of a shock and the prediction error, which were then used as predictors in the fMRI data analysis.

Specifically, we used the same simplified visual stimuli as in Batsikadze et al. (2022). Reinforcement signals $r_{t}$ for paired and non‐paired trials were coded as 1 and 0, respectively. The network architecture used to represent the agent's value function comprised two hidden fully connected layers with 64 units each and an output layer to represent the probability of the shock. For each of the two trial sequences experienced by the participants, 25 randomly initialized agents were trained. On each trial t, the agent predicted the probability of the US $v_{t}$ and a prediction error $\delta_{t}$, given the current stimulus $s_{t}$ and a reinforcement signal. At the end of each trial, an experience tuple $e_{t}=\left(s_{t},r_{t},\delta_{t}\right)$ was stored in the agent's memory for later replay (Lin 1992). The agents were trained using the backpropagation algorithm (Rumelhart et al. 1986) on batches of experiences of size b, which were sampled randomly from memory with a probability that was proportional to a priority score p:

$$P\left(e_{k}\right)=\frac{p_{k}}{\sum_{i=1}^{T}p_{i}}$$

Priority scores depended on the experiences' recency $\lambda^{\tau }$ where τ is the time passed since the experience and λ is a decay factor. Optionally, the priority could additionally depend on the magnitude of the US prediction error, i.e., $p=\left|\delta \right|\lambda^{\tau }$ (Schaul et al. 2015). The parameter RPE ∊ {Yes, No} indicates whether the replay priorities also depended on the magnitude of the US prediction error. The number of replays i was varied to control for the degree of learning in each trial.

While the previous model accounts for ABA renewal (Batsikadze et al. 2022), it cannot account for spontaneous recovery in the AAA paradigm, because, in the model, extinction in the same context overwrites the association formed during acquisition. Hence, we extended the model by an additional replay phase, which takes place between day 1 and day 2 trials and serves to recover the initially acquired association. The sleep replay phase prioritized experiences according to the reinforcement $r_{k}$ received in a trial and consisted of a total of 100 replays of batches of size 64. Reactivation probabilities for sleep replays were computed as follows:

$$P\left(e_{k}\right)=\frac{e^{\beta r_{k}}}{\sum_{i=1}^{T}e^{\beta r_{i}}}$$

where β denotes the inverse temperature and controls the relative difference of reactivation probabilities.

### 2.5 Hyper‐Parameter Fitting

We averaged SCRs from CS+ and CS‐ trials separately for each trial sequence. The averaged SCRs were accordingly defined as ${\bar{Y}}_{l}=\left({\bar{y}}_{+,1},\ldots ,{\bar{y}}_{+,N},{\bar{y}}_{-,1},\ldots ,{\bar{y}}_{-,N}\right)$, where ${\bar{y}}_{+,n}$ and ${\bar{y}}_{-,n}$ are the averaged SCRs for the n‐th CS+ and n‐th CS‐ presentations across all participants who completed a given trial sequence l, respectively. Analogously, the averaged US predictions of the model were defined as ${\bar{V}}_{l}\left(b,\lambda ,i,\beta ,\mathrm{RPE}\right)=\left({\bar{v}}_{+,1},\ldots ,{\bar{v}}_{+,N},{\bar{v}}_{-,1},\ldots ,{\bar{v}}_{-,N}\right)$, where ${\bar{v}}_{+,n}$ and ${\bar{v}}_{-,n}$ are the averaged US predictions for the n‐th CS+ and CS‐ presentations across all model instances who were trained on a given trial sequence l, respectively. The goodness of fit was defined as:

$$\bar{F}\left(b,\lambda ,i,\beta ,\mathrm{RPE}\right)=-\sum_{l=1}^{L}w_{l}\left(\left\|{\bar{Y}}_{l}-{\bar{V}}_{l}\left(b,\lambda ,i,\beta ,\mathrm{RPE}\right)\right\|+P_{\mathrm{Saf}}+P_{\mathrm{Ext}}\right)$$

where $w_{l}$ is the number of participants who experienced trial sequence l. To ensure that the overall learning curve in the model resembled that of the participants, we added the following penalty terms, if the model failed to

1. learn that CS‐ is not followed by the US: $P_{\mathrm{Saf}}=\left\{\begin{array}{c} 0,{\bar{v}}_{\mathrm{Acq},\mathrm{End}}<0.1\\ 10,{\bar{v}}_{\mathrm{Acq}.\mathrm{End}}\geq 0.1 \end{array}\right.$ where ${\bar{v}}_{\mathrm{Acq},\mathrm{End}}$ is the average US prediction over the last 4 CS‐ presentations of acquisition training.
2. successfully extinguish the CR: $P_{\mathrm{Ext}}=\left\{\begin{array}{c} 0,{\bar{v}}_{\mathrm{Ext},\mathrm{End}}<0.15\\ 10,{\bar{v}}_{\mathrm{Ext}.\mathrm{End}}\geq 0.15 \end{array}\right.$ where ${\bar{v}}_{\mathrm{Ext},\mathrm{End}}$ is the average US prediction over the last 4 CS+ presentations of extinction training.

A grid search was conducted over the hyper‐parameter sets shown in Table 1. The model with the best goodness‐of‐fit was then chosen to generate predictions that were used as parametric modulations in the fMRI data analysis.

**TABLE 1:** Modeling.

Grid search parameters	
*b* (batch size)	{1, 16, 32, 64, 96, 128}
*λ* (decay factor)	{0.05, 0.4, 0.5, 0.6, 0.7, 0.8, 0.825, 0.85, 0.895, 1}
*i* (training repeats)	{1, 2, 3, 4, 5, 6, 7, 8, 9, 10, 11, 12}
*β* (inverse temperature)	{1, 2, 3, 4, 5}
*RPE*	{Yes, No}

*Note:* Parameter values the grid search was run for.

### 2.6 Cerebellar Transcranial Alternating Current Stimulation (ctACS)

Participants were randomly assigned to either a verum or a sham stimulation group and received 2 mA (peak‐to‐peak) 6 Hz ctACS for 15 min or sham ctACS for 30 s. The final data analysis included SCR data from 22 participants [10 men/12 women] in the verum group and 18 participants [8F/10M] in the sham group, and for reasons given above, the final fMRI analysis included data from 19 participants [9F/10M] in the verum group and 16 participants [7F/9M] in the sham group. ctACS started two minutes before the start of the extinction phase and lasted until its end. To ensure good contact with the scalp and provide a conductive medium, a thin layer of Ten20 paste (Ten20, Weaver) was applied to each rubber electrode. To maintain consistency in experimental conditions, minimize stimulation‐related potential discomfort, and ensure effective blinding, a topical anesthetic cream containing 2.5% Lidocaine and 2.5% Prilocaine (EMLA, Aspen, Ireland) was administered to the skin at the intended electrode sites approximately 10–15 min before participants entered the MRI scanner on both days of the study (McFadden et al. 2011). The target electrode (5 cm x 7 cm) was placed vertically over the right cerebellar cortex. The non‐target electrode (5 cm x 7 cm) was placed over the right deltoid muscle. The current was ramped up and down for 15 s at the beginning and the end of the stimulation. The battery‐driven MRI‐compatible DC‐Stimulator Plus (neuroConn GmbH, Ilmenau, Germany) was used to deliver the stimulation. Both the experimenter and the participants were blinded to the type of stimulation. The study achieved double blinding by utilizing the stimulator's study mode, where pre‐assigned five‐digit codes were inputted into the device to initiate either the active or sham protocol.

**FIGURE 2:**
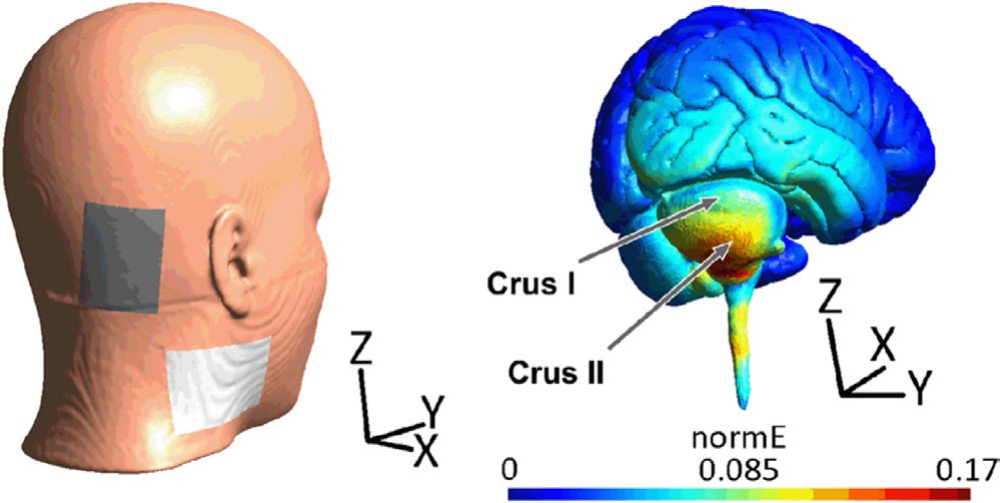
Transcranial electric stimulation (tES) montage resulting in most selective stimulation of target area, the right Crus I (neck position corresponds to shoulder position). normE: the magnitude of stimulation‐induced electric field.

We found in a prior study that cerebellar tDCS effects on neurophysiological measures (i.e., cerebellar‐brain inhibition) were not significantly different using the more standard position of the return electrode on the buccinator muscle compared to the less commonly used position on the deltoid muscle (Batsikadze et al. 2019). Thus, the deltoid muscle was used as position for the return electrode, which is preferred in the MR scanner as compared to the face reducing the risk of possible side effects. In addition, computational modeling of transcranial electric stimulation (tES) induced electric fields was performed to test which cerebellar electrode positions result in the most selective stimulation of the cerebellar target areas. Target areas were determined based on fMRI data from Ernst et al. (2019). Simulations were performed using SimNIBS (Thielscher et al. 2015) to choose a montage which maximizes the average electric field (EF) value in the right Crus I with the least possible average EF values in the vermis, left crus I, and extracerebellar areas involved in the fear network including R/L dorsolateral PFC (dlPFC), hippocampus, and amygdala. This resulted in a montage with the cerebellar electrode centered on PO10 (according to the 10–20 EEG system, (Homan 2015)) with the return electrode over the neck—as a proxy for the deltoid muscle (Figure 2). The cerebellar electrode was placed accordingly. Note that although electrode position was optimized for stimulation of Crus I, maximum stimulation is still in Crus II, which is a characteristic finding in cerebellar tDCS (Batsikadze et al. 2019; D'Mello et al. 2017).

### 2.7 MRI Acquisition

All MR images were acquired with the participants lying headfirst supine inside a clinical 3 T scanner (VIDA, Siemens Healthineers, Erlangen, Germany) equipped with a 1‐channel transmit/64‐channel receive array head coil. Electrodes for ctACS were attached to the participant's head before placement in the scanner and fixed in position with ample bandages. As needed, inflatable cushions were used to fix the head position within the coil. To minimize inter‐subject placement variance, the auto‐alignment option was used and manually corrected if needed.

Isotropic 1 mm resolution anatomic T1‐weighted images were acquired using the MPRAGE sequence immediately before functional MRI acquisition on day 1. Sequence parameters were selected as follows: TR/TE, 2530/2.27 ms, TI, 1100 ms, flip angle, 7°, parallel acceleration factor, 2, acquisition matrix, 256 × 256, number of slices, 176, TA, 6:03 min. Additionally, isotropic 1‐mm resolution MP2RAGE was acquired after fMRI acquisition on day 1. Sequence parameters were selected as follows: TR/TE, 5000/2.98 ms, TI1/TI2, 700/2500 ms, flip angles 1/2, 4°/5°, parallel acceleration factor, 3, acquisition matrix, 256 × 256, number of slices, 176, TA, 7:19 min. No anatomic images were acquired on day 2.

Functional MRI acquisition was performed to cover the whole brain with an isotropic voxel size of 2.5 mm^3^ using a fat‐saturated, two‐dimensional simultaneous multi slice echo planar image (SMS‐EPI) sequence. Further imaging parameters were selected as follows: TR/TE, 1790/30.0 ms, flip angle, 60°, parallel acceleration factor (GRAPPA), 2, SMS factor, 2, acquisition matrix, 96 × 96, number of slices, 60, bandwidth, 2170 Hz/pixel, phase encoding direction anterior to posterior. To correct for distortion artifacts before functional acquisition, two brief EPI sequences were acquired: (a) five frames of opposed‐phase reference, i.e., with opposite phase encoding direction (posterior to anterior), and (b) one frame as single band reference, i.e., without SMS acceleration, and otherwise identical parameter settings as the actual fMRI sequence.

Additionally, resting state and diffusion images were acquired immediately after fMRI acquisition on day 2. However, resting state and diffusion data are not presented in this work.

### 2.8 Image Preprocessing

DICOM data was converted to NIFTI format using dcm2niix software (version 1.0.20201102; Li et al. 2016) and all data was structured to comply with brain imaging data structure (BIDS; Poldrack et al. 2024). All evaluation steps were then performed on a laptop running 64‐bit Linux (distribution Ubuntu 22.04). Data preprocessing was carried out using the fMRIPrep Docker container (version 23.1.4), a robust preprocessing pipeline that standardizes functional MRI preprocessing (Esteban et al. 2019), with default settings and with Freesurfer surface processing disabled (‐fs‐no‐reconall). The preprocessing pipeline involved several key steps, including skull stripping, head motion correction, susceptibility distortion correction, and spatial normalization to the MNI152NLin2009cAsym template with an isotropic resolution of 2 mm.

Finally, the functional BOLD volume images were smoothed using a smoothing kernel of 6 mm, and voxel timeseries were low‐pass filtered with a Gaussian‐weighted least‐squares straight line fitting, with sigma = 2.548, as implemented in FSL Feat.

### 2.9 Analysis and Statistics

#### 2.9.1 Skin Conductance Responses (SCRs)

Skin conductance data was low‐pass filtered with a 10 Hz cutoff using a hardware filter (EDA100C‐MRI module, BIOPAC Systems Inc., Goleta, CA). Offline data processing was performed using the MATLAB‐based (Release 2019a, RRID: SCR_001622, The MathWorks) software EDA‐Analysis App (Otto et al. 2023). SCRs were identified as the maximum trough‐to‐peak amplitudes that met specific criteria, including a minimum amplitude of 0.01 μS and a minimum rise time of 500 ms (Boucsein et al. 2012), occurring within a time window of 1 to 8.5 s after the onset of the CS within the CS response window (Pineles et al. 2009) and from 8.5 to 13 s after CS onset within the unconditioned response window (Merz et al. 2014). The 8.5 s limit corresponds to the presumed onset of the response to the aversive stimulus in reinforced trials. Trials not meeting the criteria were scored as zero and included in subsequent analysis (Pineles et al. 2009).

The resulting raw SCRs were averaged into blocks and normalized through a logarithmic [LN (1 + SCR)] transformation (Boucsein et al. 2012; Venables and Christie 1980). Three habituation trials of the same CS were combined to form single blocks. In the subsequent phases, the trials of the same CS were divided into equal‐sized early and late blocks. Specifically, in the acquisition and extinction training, the averaging included the first and last eight trials of each CS, while in the recall phase, the averaging included the first and last six trials of each CS. The Shapiro–Wilk test was used to test the log‐transformed data and the distribution of residuals for normality. Since the normality test revealed a non‐normal distribution of SCRs and the residuals (*p* < 0.05), data were analyzed with non‐parametric statistical analysis for repeated measures using rank‐based F‐tests (ANOVAF option in the PROC MIXED method in SAS, SAS Studio 3.8, SAS Institute Inc., Cary, NC, USA) and the nparLD R package (http://www.R‐project.org/), which is recommended for dealing with skewed distributions, outliers, or small sample sizes (Brunner et al. 2002). These methods use an ANOVA‐type statistic with the denominator degrees of freedom set to infinity (Brunner et al. 2002; Noguchi et al. 2012) to enhance the reliability of the ANOVA‐type statistic. Using finite denominator degrees of freedom can lead to increased type I errors (Bathke et al. 2009).

Non‐parametric ANOVA‐type statistics for repeated measures (ATS) were used separately for each phase with SCRs as the dependent variable and stimulus (CS+, CS‐) and block (early, late) as within‐subjects factors and group (verum, sham) as between‐subjects factors as well as their interactions. In addition, late acquisition training and early extinction training were compared. For US‐related SCRs, ATS was conducted using a similar model but with one exception: during acquisition training, stimulus was defined as CS+/US, CS+/no‐US, and CS‐. In case of significant results of ATS, *post hoc* comparisons were performed using least square means tests and were adjusted for multiple comparisons using the Tukey–Kramer method.

To address the individual variability of SCR amplitudes, we computed the differential skin conductance responses (SCR_diff_) by subtracting the SCR to CS‐ from the SCR to CS+ for each block (Schellen et al. 2024). This enabled us to quantify the differential reaction to the conditioned stimuli. ATS was used separately for each phase, with SCR_diff_ as the dependent variable, block (early, late) as the within‐subjects factor, and group (verum, sham) as the between‐subject factor, in addition to their interactions.

To evaluate the robustness of our trial selection and minimize potential bias from block averaging, we also analyzed SCRs and SCR_diff_s at the single‐trial level across acquisition, extinction, and recall. These data were analyzed using the same non‐parametric statistical models as the block‐averaged data.

To quantify the effect sizes, we used a metric called relative treatment effects (RTE). The RTE represents the probability that a randomly selected value from one specific factor level of interest (*X*) is greater than, less than, or equal to the mean value (*Y*) of a fixed reference distribution, expressed as *px = P* (*X > Y*), *px = P* (*X < Y*), or *px = P* (*X = Y*). If *p*_*X*_ is lower than *p*_*Z*_, it suggests that measurements taken under condition *X* are generally smaller than those under condition *Z*. Conversely, *p*_*X*_ = *p*_*Z*_ indicates no consistent difference between the data from conditions *X* and *Z*. For example, a *p*_*X*_ value of 0.25 means there is approximately a 25% chance of randomly selecting a participant from the dataset who would score lower than a randomly chosen participant from condition *X* (Rubarth et al. 2022).

#### 2.9.2 Questionnaires

Questionnaires were analyzed using ATS with the respective rating as dependent variable and stimulus (CS+, CS‐) and time (prior to, post fear acquisition training, post extinction training and post recall) as within‐subjects factor and group (verum, sham) as between‐subjects factor as well as their interactions.

Side effect ratings were analyzed using ATS with the respective rating as the dependent variable, time (prior and post ctACS) as a within‐subjects factor, and group (verum, sham) as a between‐subjects factor as well as their interaction.

#### 2.9.3 fMRI Analysis

The first‐level analysis was performed on the preprocessed functional data, independently for all runs using FSL FEAT (FMRI Expert Analysis Tool) Version 6.0.1, part of FSL (FMRIB's Software Library, www.fmrib.ox.ac.uk/fsl). Feat routines were invoked from Python using Nipype (version 1.8.6). In‐scanner motion parameters estimated by fMRIprep (i.e., 3 rotations and 3 translations) were used as first‐level nuisance regressors.

The first‐level analysis was modeled as an event related‐design for the entire experiment, i.e., all event durations were set to 0 s. Onsets of presentations of the CS+, CS‐, and US (including the corresponding point in time for unpaired trials, i.e., US omission after CS presentation, further referred to as no‐US) were modeled as individual events. Individual events were blocked as shown in Figure 1. First level main effect contrasts against baseline and appropriate differential first level contrasts were generated.

Finally, a second separate first level analysis was performed on the preprocessed and smoothed functional data. For each experimental phase, all events for each event type (CS, US and no‐US) were grouped irrespective of CS trial type (CS+/CS‐). Trial‐by‐trial parameters derived from the learning model were applied as parametric modulations, i.e., the mean prediction parameters for the CS events and the mean absolute prediction error parameters for US and no‐US events.

Similarly to previous studies of our group (Batsikadze et al. 2022; Ernst et al. 2019; Nio et al. 2025), the ‘CS+ > CS‐’contrast was used to identify brain regions involved in the prediction of the US, i.e., the learned fear association. The ‘no‐US post CS+ > no‐US post CS‐‘contrast focused on expectancy violation, i.e., the unexpected omission of the aversive US in unreinforced CS+ trials during acquisition training (using partial reinforcement), initial extinction trials and recall trials involving spontaneous recovery. The resulting prediction error signals are believed to drive the updating of previously learned threat associations, thereby leading to extinction learning. In addition, a parametric modulation analysis was conducted to examine how brain activity varied in relation to trial‐by‐trial differences in prediction and prediction error.

For second level analysis, first‐level contrasts were tested with a fixed‐effect analysis within, across, and between groups. Cluster‐wise correction for multiple comparisons was applied using a Z‐threshold of 3.1 and a cluster significance threshold of *p* < 0.05 (Worsley 2001). The anatomical brain locations of significant clusters were determined using the Harvard‐Oxford cortical and subcortical structural atlases (https://identifiers.org/neurovault.collection:262) and Cerebellum atlas (the version normalized with FLIRT). When the Harvard‐Oxford atlas was insufficient for identification of subcortical structures, manual identification was conducted using the multi‐contrast anatomical subcortical structures (MASSP) atlas (Bazin et al. 2020). The correspondence between anatomical and functional areas was verified by author G.B. To display results, cerebellar activation maps were plotted on cerebellar flatmaps (A spatially unbiased atlas template of the cerebellum and brainstem [SUIT] space; Diedrichsen 2006; Diedrichsen and Zotow 2015).

Furthermore, we added an analysis of effect sizes. Specifically, voxel‐wise maps (Cohen's *d* effect size maps) were created for each group (sham and verum) and for the group comparison (verum vs. sham) separately for acquisition training, extinction training, and recall. For each contrast (‘CS+ > CS‐’ and ‘no‐US post CS+ > no‐US post CS‐’), Cohen's *d* was calculated by dividing the contrast image (COPE) by the residual standard deviation at each voxel. The residual standard deviation was taken from the 4D residual image of the general linear model, using the standard deviation across time. This was done separately for each second‐level analysis for group maps and group differences. The resulting Cohen's d maps were shown with a color scale from minimum to maximum. To highlight statistically significant effects, thresholded z‐statistic maps (*p* < 0.05) from the second‐level analysis were overlaid as black outlines on the effect size maps. Cohen's *d* values and their approximate 95% confidence intervals are provided in the corresponding tables (Tables S4–S7).

Finally, mean *β* values were extracted for six VOIs from first‐level *β* maps (contrasted against rest) for each participant. VOIs were chosen based on the meta‐analysis of fMRI studies on fear extinction performed by Fullana et al. (2018). They found that the anterior insular cortex, anterior cingulate cortex (ACC), but also the cerebellum, most consistently showed fMRI activation related to fear extinction and recall (in studies using a ‘CS+ > CS‐’ contrast). In our data, these regions were also active in a minimum conjunction analysis combining the ‘CS+ > CS‐’ and ‘no‐US post CS+ > no‐US post CS‐’ contrasts during fear acquisition training. The conjunction analysis was performed using the *easythresh_conj* script (Nichols et al. 2005) in FSL, with a whole‐brain mask and a threshold of z > 3.1. We then masked the resulting thresholded conjunction map using regions defined in the Automated Anatomical Labeling Atlas 3 (AAL3; Rolls et al. 2020) for the left and right cerebellum, ACC, and insulae separately, setting all values outside these regions to zero. This resulted in six VOIs: left and right cerebellum, left and right ACC, and left and right insulae, each overlapping with the conjunction map. ATS were performed separately for each VOI and each response window (CS and no‐US), using respective mean *β* values as the dependent variable. Stimulus (CS+, CS‐) and block (early, late) were included as within‐subjects factors, and group (verum, sham) as a between‐subjects factor as well as their interactions.

#### 2.9.4 Use of Artificial Intelligence Tools

OpenAI GPT‐5 was used exclusively for language and phrasing. The authors reviewed and edited all content and are fully responsible for the final manuscript.

## 3 Results

### 3.1 CS Related Skin Conductance Responses (SCRs)

#### 3.1.1 Habituation Phase (Day 1)

There was no significant difference in the mean SCR amplitudes between the CS+ and CS‐. Additionally, there was no group difference in differential SCRs (SCR_diff_). ATS did not reveal any significant effects or interactions (all *p* ≥ 0.131, Table S1).

#### 3.1.2 Acquisition Training (Day 1)

**FIGURE 3:**
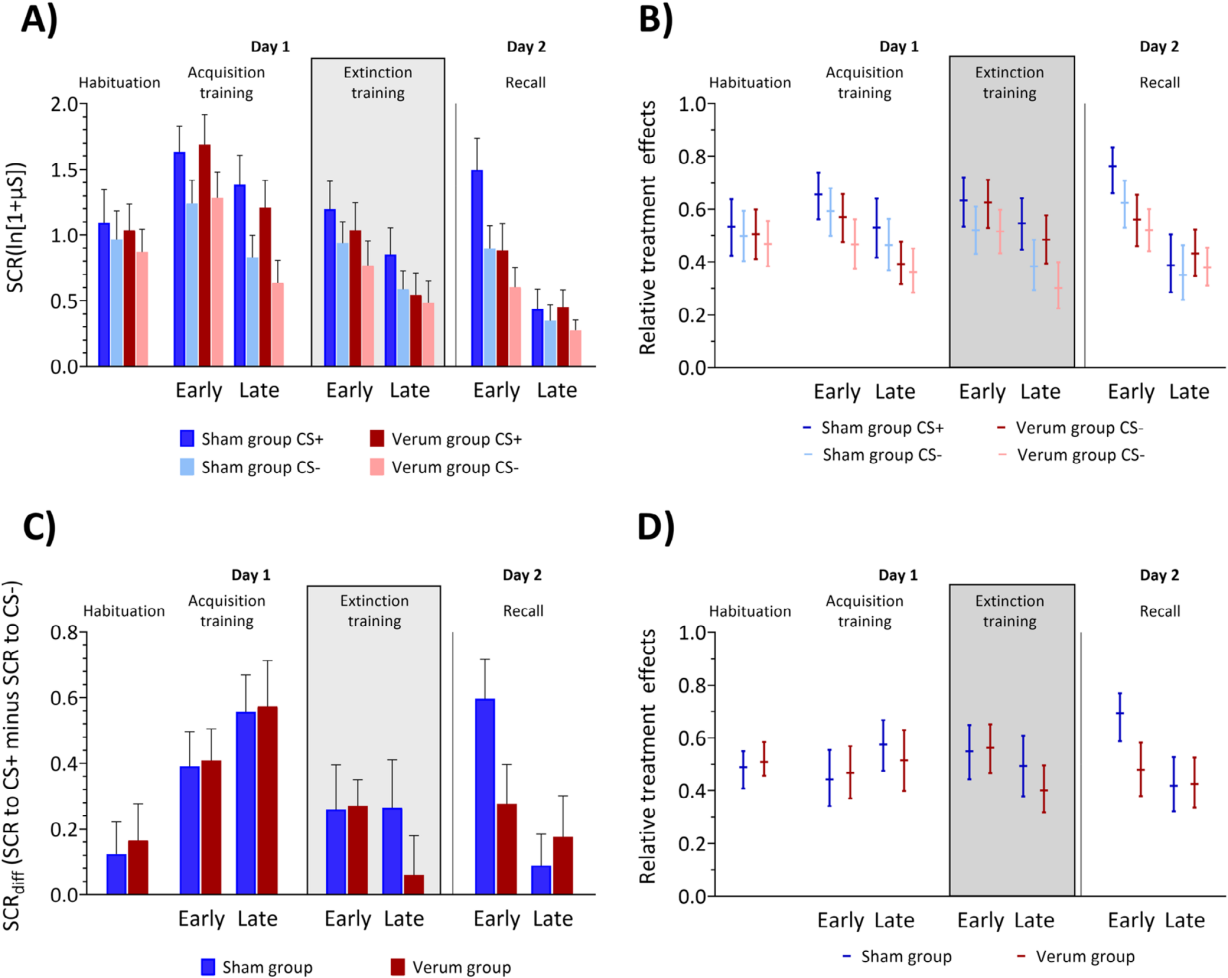
Skin conductance response (SCR) amplitudes and respective relative treatment effects (RTEs). (A) CS related SCRs, (C) CS related differential skin conductance responses (SCR_diff_), (E) US/no‐US related SCRs and (B, D, F) corresponding RTE estimates during habituation, fear acquisition training, extinction training and recall. (A, C, E) Colored bars represent group mean (log‐transformed) values for habituation, early and late blocks of fear acquisition training, extinction training and recall. Error bars indicate S.E.M. (B, D, F) Horizontal lines denote median RTEs and whiskers denote 95% confidence intervals. Blue colors = sham group, red colors = verum group. Dark colors: CS+, light colors: CS‐. Striped bars in panel (E) represent SCRs in the paired CS+/US trials (i.e., response to the aversive US), while solid bars represent SCRs to the omission (in CS+/no‐US trials) or absence (in CS‐ trials) of the US. The lightning symbol in panel (F) represents RTEs of the paired CS+/US trials. The grey background in the figure marks the period during which transcranial alternating current stimulation (ctACS) was administered.

Both groups of participants showed higher SCRs in response to the CS+ than to the CS‐, and the responses were larger during the early block compared to the late block. The differences between the CS+ and CS‐ responses were significant for both groups (Figure 3). ATS revealed significant main effects of Block (*F*_1_ = 59.53, *p* < 0.001), Stimulus Type (*F*_1_ = 55.00, *p* < 0.001), and a significant Stimulus Type × Block interaction (*F*_1_ = 5.00, *p* = 0.025; Table S1). No significant group differences or interactions were revealed (all *p* ≥ 0.081). *Post hoc* exploratory analysis of the Stimulus Type × Block differences revealed significantly higher SCRs in the early vs. late acquisition blocks in both groups towards both CSs, and significantly higher SCRs towards CS+ vs. CS‐ in both early and late blocks (all *p* < 0.001, least squares means test).

The SCR_diff_ analysis did not show any significant effects of Block, Group, or their interaction (all *p* ≥ 0.085).

#### 3.1.3 Extinction Training (Day 1)

Both groups showed a difference of SCR amplitudes between the CS+ and CS‐, with higher SCRs elicited by the CS+. Larger SCRs were observed in the early block compared to the late block (Figure 3). ATS revealed a significant main effect of Block (*F*_1_ = 34.25, *p* < 0.001) and Stimulus Type (*F*_1_ = 13.41, *p* < 0.001). No significant group differences and interactions were revealed (all *p* ≥ 0.167; Table S1).

The SCR_diff_ analysis showed a significant Block effect (*F*_1_ = 5.41, *p* = 0.02). SCR_diff_ values were significantly higher in the early block compared to the late block (*p* = 0.026, least square means test). The Group main effect and Block × Group interaction were not significant (both *p* ≥ 0.262; Table S1).

Visual inspection of Figure 3A,C suggest that, in late extinction, extinction effects were stronger in the verum group compared to the sham group. This was, however, not reflected in the statistical analysis (i.e., no significant Group effects, and none of the interactions involving Group coming even close to significance). Exploratory analysis of the late extinction block showed no significant Group effect (*p* = 0.134) and no significant Stimulus Type × Group interaction (*p* = 0.689). For differential SCRs, the Group effect in late extinction was also not significant (*p* = 0.382).

#### 3.1.4 Recall (Day 2)

In both groups, the SCRs were higher in the early compared to the late block. In the early recall phase, the SCRs related to the CS+ were larger than to the CS‐ in the sham group, but this was not observed in the verum group (Figure 3). In the late recall phase, both the sham and verum groups responded to both the CS+ and CS‐ in a similar manner. ATS revealed significant main effects of Block (*F*_1_ = 72.31, *p* < 0.001), Stimulus Type (*F*_1_ = 9.03, *p* = 0.003), and a significant Block × Group (*F*_1_ = 12.16, *p* < 0.001) interaction. No other significant main effects or interactions were revealed (all *p* ≥ 0.190; Table S1). The significant interaction between Block and Group was driven by a disordinal interaction, with the SCRs in the early sham block being larger than those in the late verum block (*p* = 0.002, least square means test).

Exploratory *post hoc* ATS on the first recall block revealed a significant main effect of Stimulus Type (*F*_1_ = 18.84, *p* < 0.001) and Stimulus Type × Group interaction (*F*_1_ = 4.05, *p* = 0.044). The effect of Group came close to significance (*F*_1_ = 3.68, *p* = 0.055). The sham group showed significantly higher SCRs related to the CS+ compared to the CS‐ in pairwise comparisons (least square means test, *p* < 0.001), while the verum group did not (least square means test, *p* = 0.516; Figure 3).

Regarding SCR_diff_, ATS revealed a significant main effect of Block (*F*_1_ = 10.35, *p* < 0.001) and a significant interaction between Block and Group (*F*_1_ = 4.63, *p* = 0.031; Table S1). SCR_diff_ values were significantly higher in the early block compared to the late block (*p* = 0.003, least square means test); however, this effect (early vs. late block) was only present in the sham (*p* = 0.002, least square means test), but not in the verum group (*p* = 0.890, least squares means test). Additionally, exploratory ATS on the first recall block showed a significant main effect of Group (*F*_1_ = 4.83, *p* = 0.028), with the sham group exhibiting significantly higher differential SCRs.

Analyses of US vs. no‐US related SCRs, as well as questionnaire data, are provided in the (Figures S1, S3, and Table S1).

Detailed visualization of trial‐by‐trial SCRs and differential SCRs is presented in Figure S2. Results of the ATS are provided in Table S3.

### 3.2 fMRI Data

As stated above, the anatomical brain locations were determined using the Harvard‐Oxford atlas for cortical and subcortical structures (https://identifiers.org/neurovault.collection:262) the Cerebellum atlas (the version normalized with FLIRT), and the MASSP atlas (Bazin et al. 2020).

### 3.3 Activations During Fear Acquisition Training

**FIGURE 4:**
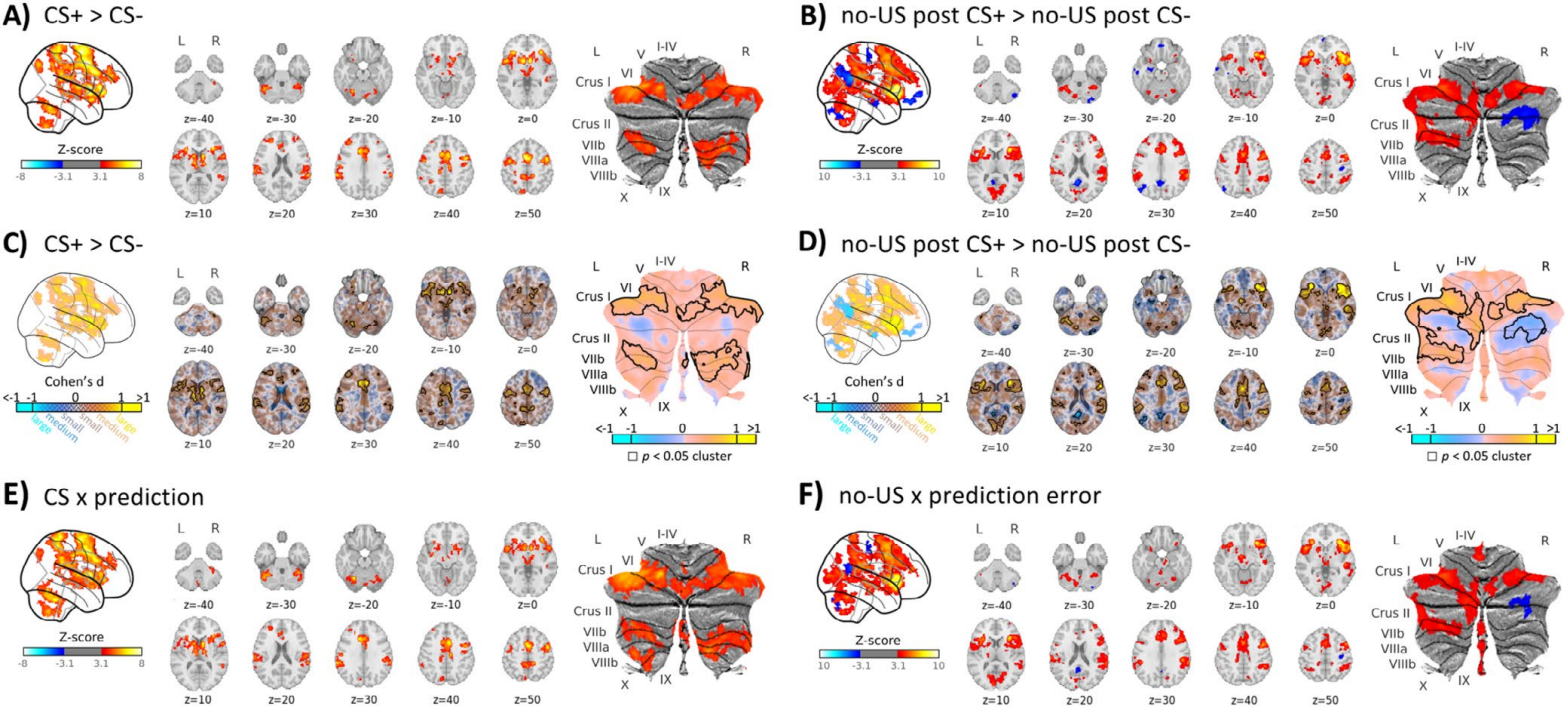
Cerebral and cerebellar activations and respective effect size maps (Cohen's *d*) during fear acquisition training related to the (A, C) presentation of the CSs (contrast ‘CS+ > CS‐’) and (B, D) omission of the aversive US (contrast ‘no‐US post CS+ > no‐US post CS‐’), (E) Parametric modulation of CS events with individual mean prediction values (CS × prediction) and (F) parametric modulation of omission of US events at CS termination with individual absolute mean prediction error values (no‐US × prediction error). (A, B) contrasts ‘no‐US post CS+ > no‐US post CS‐’ and ‘CS+ > CS‐’ are shown in red and contrasts ‘no‐US post CS+ < no‐US post CS‐’ and ‘CS+ < CS‐’ are shown in blue. (C, D, E, F) Red colors indicate increased activation, and blue colors indicate decreased activation. Black frames on panels C and D indicate clusters that reached statistical significance at *p* < 0.05. CS: conditioned stimulus; L: left; R: right; SUIT spatially unbiased atlas template of the cerebellum; results of fMRI analysis are provided in Tables S4 and S6.

**FIGURE 5:**
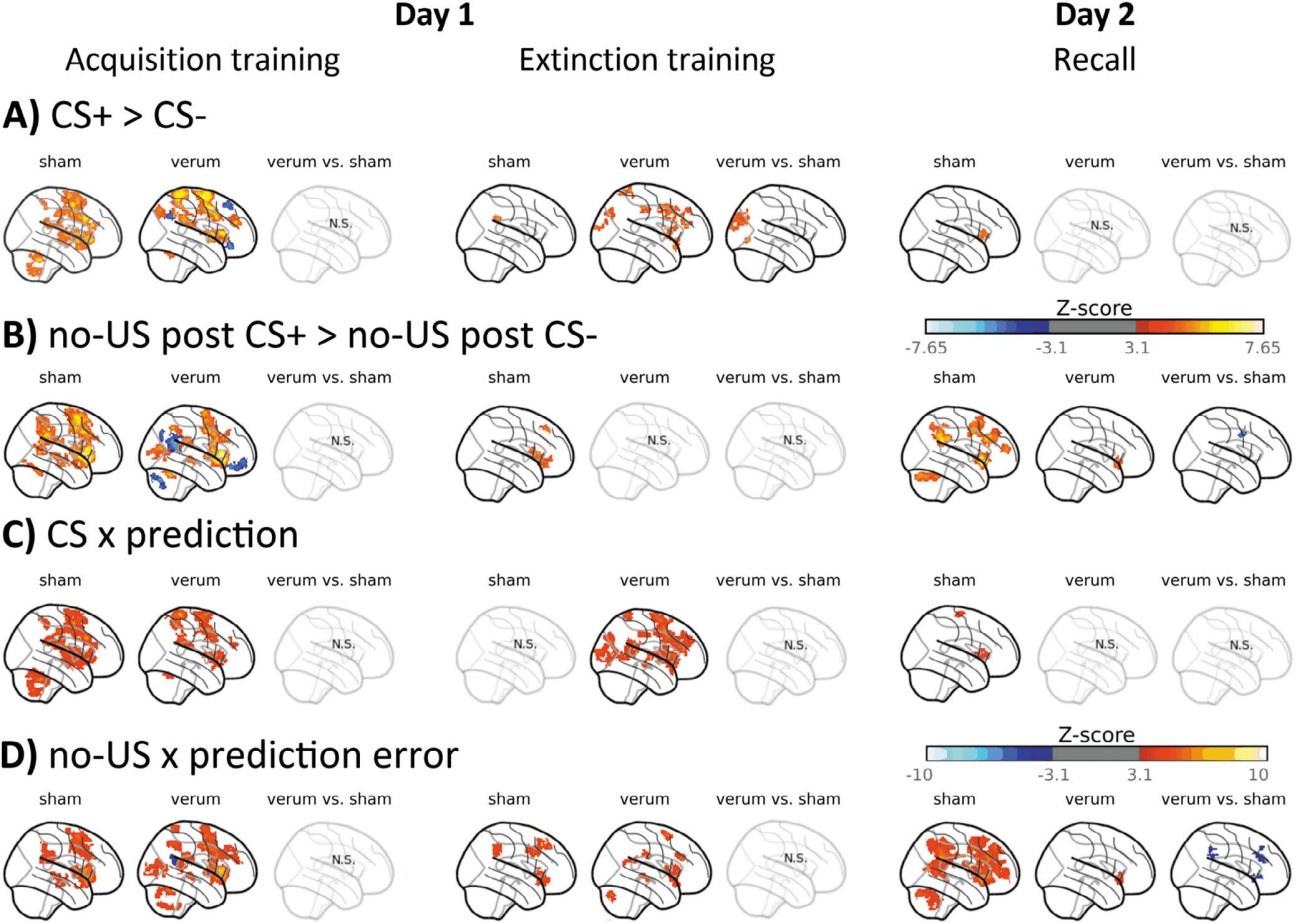
The glass brain visualization showing cerebral and cerebellar activations during fear acquisition training, extinction training and recall related to the (A) prediction of the CS (contrast ‘CS+ > CS‐’), (B) omission of the aversive US (contrast ‘no‐US post CS+ > no‐US post CS‐’) provided separately for verum and sham groups alongside a differential contrast (contrast ‘verum vs sham’), (C) Parametric modulation of CS events with individual mean prediction values (CS × prediction) and (D) parametric modulation of omission of US events at CS termination with individual absolute mean prediction error values (no‐US × prediction error). (A, B). In the individual groups, contrasts ‘no‐US post CS+ > no‐US post CS‐’ and ‘CS+ > CS‐’ are shown in red and contrasts ‘no‐US post CS+ < no‐US post CS‐’ and ‘CS+ < CS‐’ are shown in blue. (C, D) In the individual group contrasts, increases are shown in red and decreases in blue. For the differential contrast ‘verum vs sham’, shades of blue denote higher activation in the sham group, while shades of red denote higher activation in the verum group. CS:conditioned stimulus; US: unconditioned stimulus; N.S. – no significant clusters. Results of fMRI analysis are provided in Tables S4, S5, S6, and S7.

**FIGURE 6:**
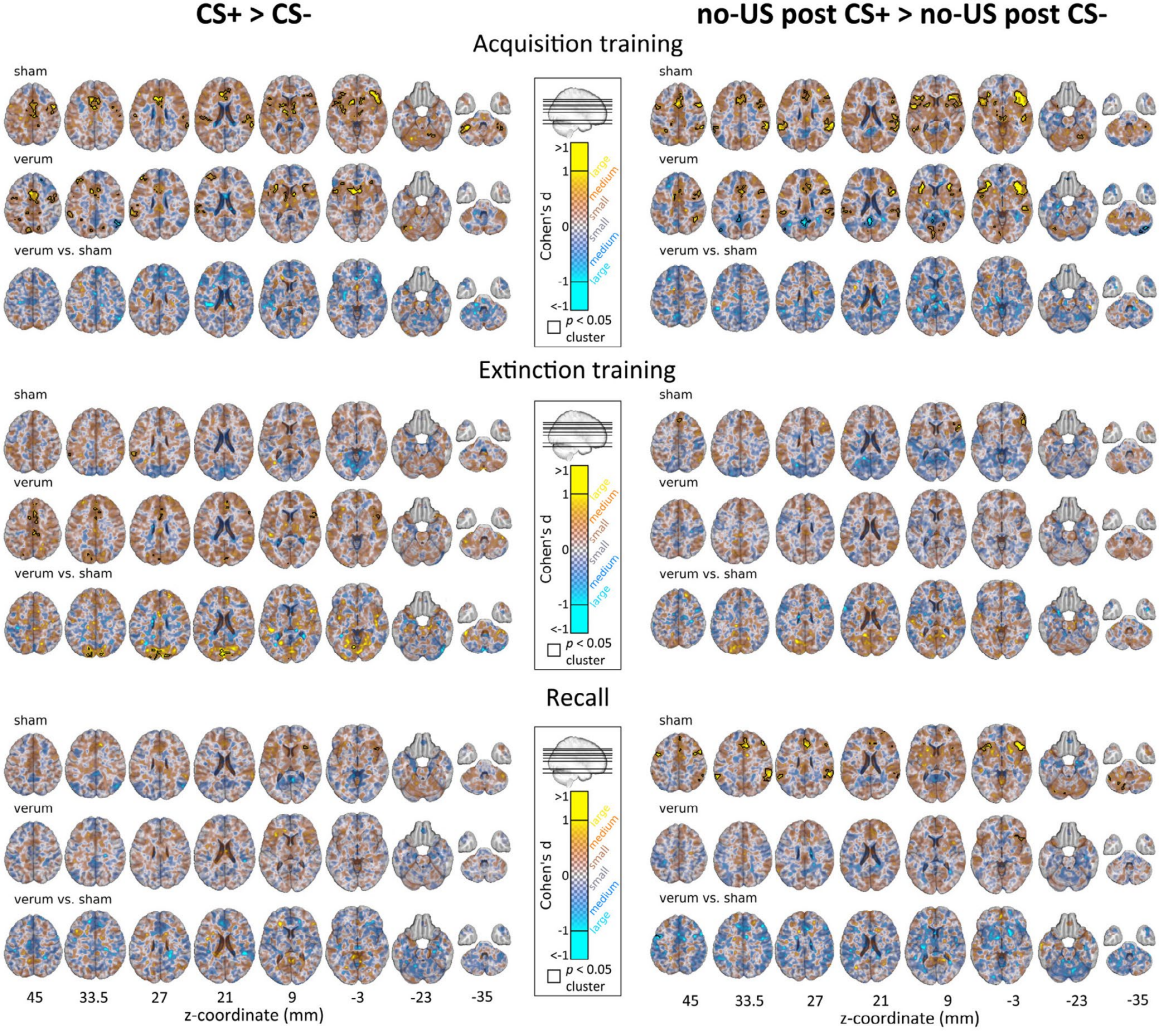
Effect size maps (Cohen's *d*) for the contrasts ‘CS+ > CS‐’ (left column) and ‘no‐US post CS+ > no‐US post CS‐’ (right column), shown across acquisition training, extinction training, and recall. Each phase displays results for the sham and verum groups separately, as well as for the direct group comparison (verum vs. sham). For the within‐group contrasts (sham and verum), red/yellow indicates increased activation, and blue indicates decreased activation. In the verum vs. sham comparison, red/yellow represents verum > sham, and blue represents sham > verum. Black frames indicate clusters that reached statistical significance at *p* < 0.05. z‐coordinates (in mm) are indicated below each axial slice.

Because ctACS was administered during extinction training, no significant differences were expected between the sham and verum groups during acquisition training. As anticipated, activation patterns showed similar results for each group when analyzed separately (see Supplements). No significant between‐group differences [contrasts ‘verum>sham’ and ‘verum<sham’] were found; therefore, findings are reported by combining participants from both groups (Figures 4, 5, 6 and Table S4).

#### 3.3.1 Activation Related to the Prediction of the Aversive Stimulus [Contrast ‘CS+ > CS‐’]

Significantly higher brain activity was observed for the CS+ compared to the CS‐ in brain areas well known to show increased differential activations during fear acquisition training (e.g., Fullana et al. 2016), including the insular cortex, ACC, frontal and parietal cortical areas, subcortical regions, midbrain, and cerebellum, specifically, bilaterally in cerebellar lobules VI (including vermis) and Crus I, as well as VII and VIII (Figure 4A shades of red, Table S4, for effect sizes see Figure 4C).

#### 3.3.2 Activation Related to the Omission of the Aversive Stimulus [Contrast ‘no‐US Post CS+ > no‐US Post CS‐’]

The pattern of differential activations related to the (unexpected) omission of the US showed overlap with areas related to the prediction of the US. Overall, activated cortical areas were more extended, and in addition, areas within the occipital lobe showed increased activations likely because attention is redirected to the CS+. Significant activations were observed in the insular cortex, ACC, frontal, parietal, occipital, subcortical, and cerebellar (left cerebellar lobules VI‐VII, left Crus I and II, right VI, and right Crus I) regions (Figure 4B, shades of red, Table S4, for effect sizes see Figure 4D).

#### 3.3.3 Activations During Fear Extinction Training

##### 3.3.3.1 Activation Related to the Prediction of the Aversive Stimulus [Contrast ‘CS+ > CS‐’]

In the sham group, activations were limited to the supramarginal gyrus, whereas in the verum group, activations were more widespread and involved insular, cingulate, frontal, parietal, and occipital regions (Figure 5A, shades of red; Table S5; for effect sizes see Figure 6). Between‐group comparisons revealed significantly higher activation in occipital regions in the verum compared to the sham group (Figure 5A, shades of red; Table S5; for effect sizes see Figure 6).

##### 3.3.3.2 Activation Related to the Omission of the Aversive Stimulus [Contrast ‘no‐US Post CS+ > no‐US Post CS‐’]

Activations were observed in frontal cortical regions in the sham group, whereas no significant activations were observed in the verum group. Between‐group differences were not significant (Figure 5B, shades of red, Table S5, for effect sizes see Figure 6).

### 3.4 Activations During Recall

#### 3.4.1 Activation Related to the Prediction of the Aversive Stimulus [Contrast ‘CS+ > CS‐’]

In the sham group, activations were observed in the insular cortex and frontal operculum (Figure 5A, shades of red, Table S5, for effect sizes see Figure 6). No significant activations were observed in the verum group, and between‐group differences were not significant [contrasts ‘verum > sham’ and ‘verum < sham’].

#### 3.4.2 Activation Related to the Omission of the Aversive Stimulus [Contrast ‘no‐US Post CS+ > no US Post CS‐’]

**FIGURE 7:**
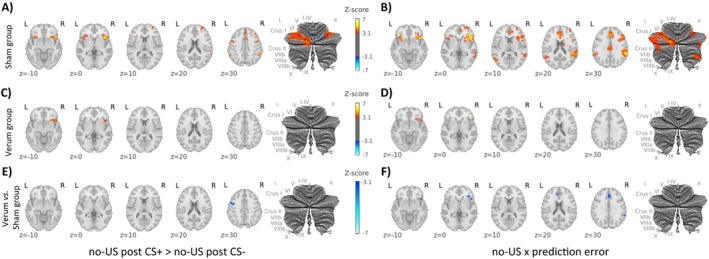
Cerebral and cerebellar activations during recall related to the omission of the aversive US (contrast ‘no‐US post CS+ > no‐US post CS‐’) and parametric modulation with learning model‐derived omission of US events at CS termination with individual absolute mean prediction error values in (A, B) the sham group, (C, D) the verum group and (E, F) for the comparison between verum and sham groups (contrast ‘verum vs. sham’). In the individual groups, contrast increases are shown in red and decreases in blue. In the differential contrast ‘verum vs sham’, shades of blue denote higher activation in the sham group, while shades of red denote higher activation in the verum group. CS: conditioned stimulus; L: left; R: right; SUIT: spatially unbiased atlas template of the cerebellum; Results of the fMRI analysis are provided in Tables S5 and S7.

During recall, widespread activations were observed in the sham group, involving insular, cingulate, frontal, parietal, occipital, subcortical, and cerebellar (bilaterally in lobules VI and Crus I) regions (Figures 5B and 7A, shades of red, Table S5, for effect sizes see Figure 6), whereas activations in the verum group were more limited and mainly involved insular and frontal regions (Figures 5B and 7C, shades of red, Table S5). Between‐group comparisons revealed significantly higher activation in the precentral gyrus (M1) in the sham compared to the verum group (Figures 5B and 7E, shades of red, Table S5).

#### 3.4.3 Parametric Modulation With Model Predictions for Shock Probability and Prediction Errors

Parametric modulation was used to test whether learning‐model derived predictions and prediction errors were associated with fMRI signal alterations during CS and no‐US events. CS events denote the onset of conditioned stimuli, while no‐US events represent instances where the US was anticipated in reinforced CS+ trials but was absent in unreinforced CS+ and CS‐ trials. Thus, the modulation is determined by trial‐by‐trial model‐derived predictions for CS events and prediction errors for no‐US events.

### 3.5 Fear Acquisition Training

No significant between‐group differences for parametric modulation effects for CS and no‐US events [contrasts ‘verum > sham’ and ‘verum < sham’] were found (Figure 5C, Table S6). Therefore, findings are reported by combining participants from both groups.

During fear acquisition training, parametric modulation effects for CS and prediction values revealed widespread significant clusters in insular, cingulate, frontal, parietal, temporal, subcortical, and cerebellar (bilaterally in lobules VI‐IX and Crus I‐II) regions (Figures 4C and 5C, shades of red, Table S6).

Parametric modulation effects for no‐US and prediction error values revealed significant activation clusters in the ACC, insular cortex, frontal, parietal, occipital, and temporal cortical areas, as well as limbic, brainstem, and cerebellum (bilaterally in lobules I‐IV and Crus I, left VII‐VIII and Crus II, vermal VIII‐IX; Figures 4D and 5D, shades of red, Table S6).

### 3.6 Fear Extinction Training

During fear extinction training, parametric modulation effects for CS prediction values were observed only in the verum group and involved the insular cortex, ACC, frontal, parietal, occipital, and temporal cortical areas as well as subcortical regions (Figure 5C, shades of red, Table S7).

Parametric modulation effects for no‐US and prediction error values revealed significant activation clusters for the sham group in frontal and parietal cortical regions (Figure 5D, Table S7, shades of red). In the verum group, significant activation clusters were found in the insular, cingulate, frontal, parietal, and temporal cortical regions (Figure 5D, shades of red, Table S7).

No significant between‐group differences for parametric modulation effects for CS and no‐US events (contrasts ‘verum > sham’ and ‘verum < sham’) were found.

### 3.7 Recall

In the recall condition, parametric modulation effects for CS and prediction values revealed significant clusters for the sham group in the insular and frontal cortical regions, whereas no significant clusters were revealed in the verum group and no significant between‐group differences were found (Figure 5C, shades of red, Table S7).

Parametric modulation effects for no‐US and prediction error values revealed significant activation clusters for the sham group in the ACC, insular, frontal and parietal cortical areas as well as bilaterally in cerebellar lobules VI‐VII and Crus I‐II, and right VIII (Figures 5D and 7B, shades of red, Table S7). In the verum group, significant activation clusters were found in the frontal and temporal cortical areas (Figures 5D and 7D, shades of red, Table S7). Between‐group comparisons revealed significantly higher activation in the sham compared to the verum group in ACC, frontal, and parietal regions (Figures 5D and 7F, shades of blue, Table S7).

### 3.8 VOI Analysis of *β* Values Related to the Prediction of the Aversive Stimulus

#### 3.8.1 Acquisition Training (Day 1)

**FIGURE 8:**
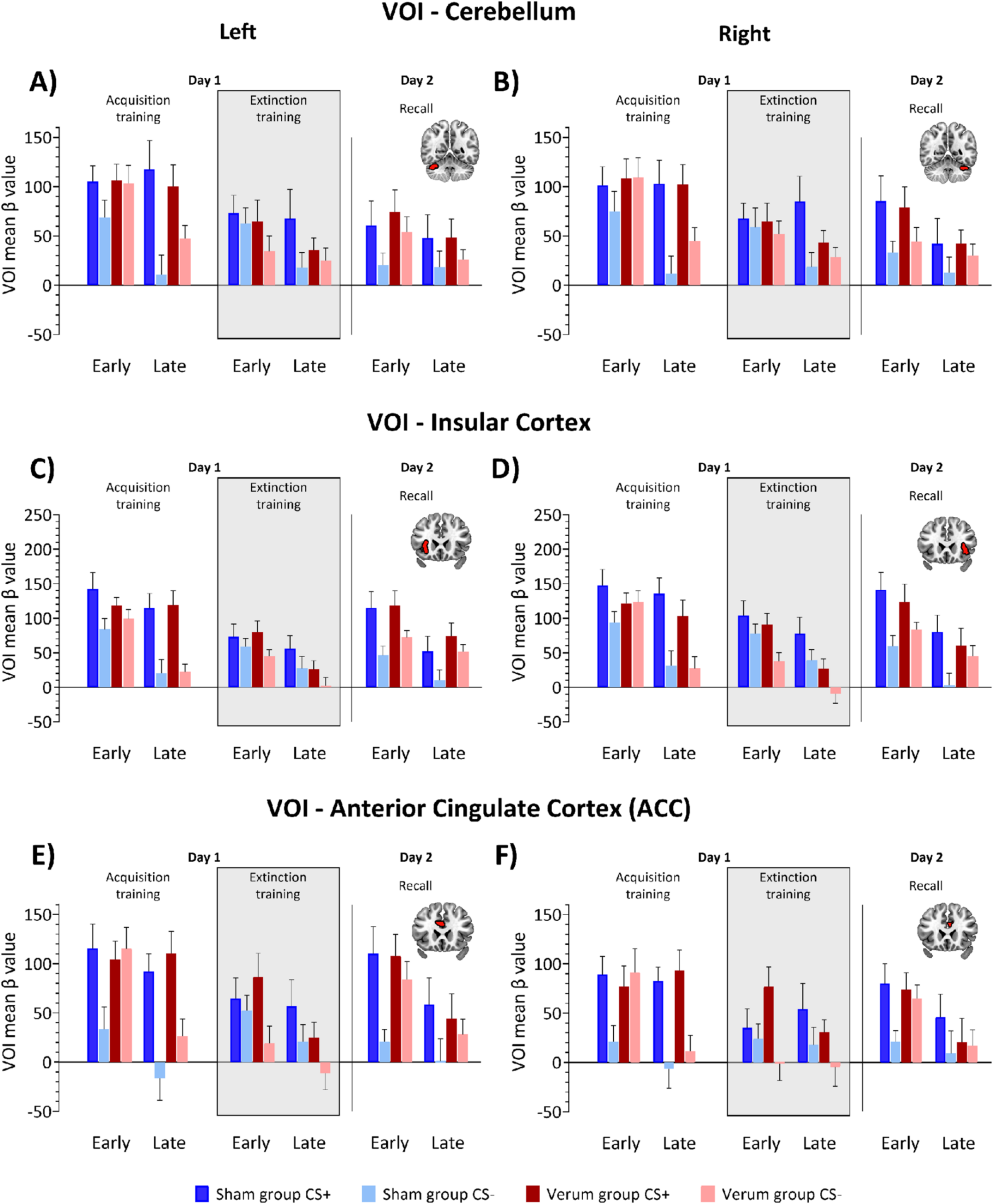
Mean *β* values related to the prediction of the aversive stimulus, extracted from six volumes of interest (VOIs). (A, B) cerebellum (lobule VI and Crus I); (C, D) insular cortex; and (E, F) anterior cingulate cortex (ACC) on day 1 (fear acquisition and extinction training) and day 2 (recall) shown across blocks. Panels A, C, and E display *β* values extracted from the left hemisphere; panels B, D, and F show values from the right hemisphere. Dark colors represent CS+, light colors represent CS‐. Blue denotes the sham group; red denotes the verum group. The grey background in the figure marks the period during which transcranial alternating current stimulation (ctACS) was administered. The VOIs in the left and right cerebellum, insular cortex and anterior cingulate cortex (ACC) are illustrated in the inserts. Respective relative treatment effect estimates are provided on (Figure S4).

*β* values were larger in response to CS+ than CS‐ across all ROIs (right/left cerebellum, right/left insulae, right/left ACC; ATS significant main effect of Stimulus Type: all *p* < 0.001, Figure 8, for effect sizes see Figure S4) and were higher in the early than the late block in cerebellum, insulae, and left ACC (ATS significant main effect of Block: all *p* ≤ 0.016, Figure 8), but not in right ACC (*p* = 0.109). Stimulus Type × Block interactions were present in every ROI (all *p* ≤ 0.004). There was no Group main effect (all *p* ≥ 0.145). A Stimulus Type × Group interaction was found in both ACC ROIs (both *p* ≤ 0.034), and a Stimulus Type × Block × Group interaction in right ACC (*p* = 0.043, Table S8).

#### 3.8.2 Extinction Training (Day 1)

*β* values were larger in response to CS+ than CS‐ across all ROIs (ATS significant main effect of Stimulus Type: all *p* ≤ 0.018). Responses were also higher in the early than the late block in both insulae and left ACC (significant main effect of Block: all *p* ≤ 0.027, Table S8, Figure 8, for effect sizes see Figure S4), but not in cerebellum or right ACC (both *p* ≥ 0.072). A Group main effect was observed in right insula (*p* = 0.018), with higher responses in sham compared to verum group, but not in other ROIs (all *p ≥* 0.119). Stimulus Type × Block interaction was present in right cerebellum only (*p* = 0.002). Stimulus Type × Block × Group interactions were observed in right cerebellum and right ACC (both *p* ≤ 0.023). No other interactions were significant (all *p ≥* 0.178).

#### 3.8.3 Recall (Day 2)

*β* values were larger in response to CS+ than CS‐ in right cerebellum, bilateral insulae and bilateral ACC (ATS significant main effect of Stimulus Type: all *p* ≤ 0.025), but not in left cerebellum (*p* = 0.057). Responses were higher in the early than in the late block in right cerebellum, bilateral insulae, and bilateral ACC (ATS significant main effect of Block: all *p ≤* 0.021, Table S8, Figure 8, for effect sizes see Figure S4), but not in left cerebellum (*p* = 0.193). A Stimulus Type × Group interaction was found in right insula (*p* = 0.025); no other interactions were significant (all *p* ≥ 0.088).

### 3.9 VOI Analysis of *β* Values Related to the Omission of the Aversive Stimulus Type

#### 3.9.1 Acquisition Training (Day 1)

**FIGURE 9:**
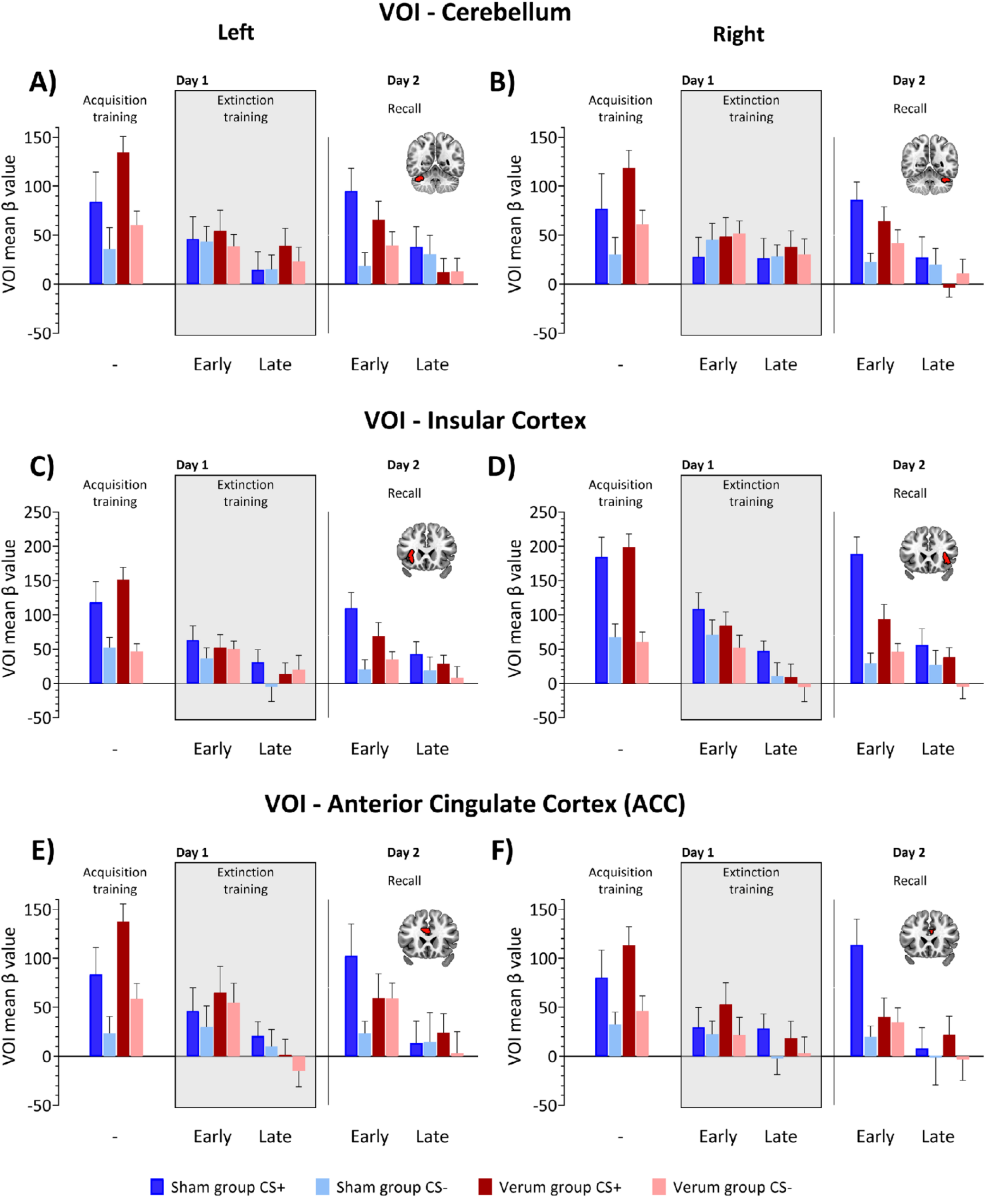
Mean *β* values related to the omission of the aversive stimulus, extracted from six volumes of interest (VOIs). (A, B) cerebellum (lobule VI and Crus I); (C, D) insular cortex; and (E, F) anterior cingulate cortex (ACC) on day 1 (fear acquisition and extinction training) and day 2 (recall) shown across blocks. Panels A, C, and E display *β* values extracted from the left hemisphere; panels B, D, and F show values from the right hemisphere. Dark colors represent CS+, light colors represent CS‐. Blue denotes the sham group; red denotes the verum group. The grey background in the figure marks the period during which transcranial alternating current stimulation (ctACS) was administered. The VOIs in the left and right cerebellum, insular cortex and anterior cingulate cortex (ACC) are illustrated in the inserts. Respective relative treatment effect estimates are provided on (Figure S5).

Across all ROIs, *β* values were higher in CS+ than in CS‐ trials (ATS significant main effect of Stimulus Type: all *p <* 0.001, Table S8, Figure 9, for effect sizes see Figure S5). No main effect of Group or any significant interaction was found (all *p ≥* 0.140).

#### 3.9.2 Extinction Training (Day 1)

In left cerebellum, both insulae and left ACC, *β* values were higher in the early compared to the late block (ATS significant main effect of Block: all *p* ≤ 0.023). In right insula, *β* values were higher for CS+ than CS‐ (ATS significant main effect of Stimulus Type: *p* = 0.015, Table S8, Figure 9, for effect sizes see Figure S5). No significant effects were observed in right cerebellum or right ACC (all *p ≥* 0.056). No Group effects or interactions were found in any ROI (all *p* ≥ 0.198, Table S8).

#### 3.9.3 Recall (Day 2)

*β* values were higher in CS+ than in CS‐ trials and higher in the early than the late block in left cerebellum, both insulae and right ACC (ATS significant main effects of Stimulus Type and Block: all *p* ≤ 0.027). In right cerebellum and left ACC, only a significant Block effect was present (both *p* < 0.001, Table S8, Figure 9, for effect sizes see Figure S5). Stimulus Type × Block interactions were observed in both right and left cerebellum and right insula (all *p* ≤ 0.032). A Stimulus Type × Block × Group interaction was found in right insula (*p* = 0.023). No significant Group effect or other interactions were significant (all *p* ≥ 0.079, least squares means test).

*Post hoc* analysis of the Stimulus Type × Block interaction showed the following: in right and left cerebellum and right insula, CS+ early > CS+ late and CS+ > CS‐ in the early block (both *p ≤* 0.003). *Post hoc* analysis of the Stimulus Type × Block × Group interaction in right insula revealed that early CS+ > early CS‐ and CS+ early > CS+ late were present in sham (both *p* ≤ 0.016) but absent in the verum group (both *p* ≥ 0.403, Figure 9, for effect sizes see Figure S5).

## 4 Discussion

Cerebellar tACS at 6 Hz during extinction training reduced spontaneous fear memory recovery. Specifically, after extinction training on day 1, testing recall of the extinguished fear association on day 2 showed that participants who had received sham ctACS exhibited a return of differential SCRs when comparing the CS+ and CS‐, which was absent in the verum group. Increased cerebellar theta activity seems to stabilize extinction memory and/or prevent the recall of the initial fear association after successful extinction training.

The present results in humans are in good agreement with findings in the animal eyeblink conditioning literature. In guinea pigs spontaneous cerebellar and medial prefrontal cortex theta (4–7 Hz) activity has been linked to successful extinction of conditioned eyeblink responses (Y. J. Wang et al. 2014). Furthermore, a decrease in cerebellar theta activity following CS exposure predicted the spontaneous reappearance of previously extinguished eyeblink responses (Wang et al. 2019). Thus, increased cerebellar theta activity seems to support extinction learning and prevent the recall of the initial fear association after successful extinction training. Wang et al. (2014) and Wang et al. (2019) have studied eyeblink conditioning, which involves the conditioning of a specific aversive response (Thompson et al. 1987). The present findings extend their observation to the extinction learning of unspecific aversive fear responses in humans. fMRI data provides some initial explanation of how.

During extinction training, the verum group exhibited stronger occipital activation, including the lateral occipital cortex, cuneus, lingual gyrus, and occipital pole, compared to the sham group related to the presentation of the CS+ (‘contrast CS+ > CS‐‘). Cortical plasticity in the visual cortex has been demonstrated to contribute to both the acquisition and extinction of conditioned fear (Petro et al. 2017; Xie et al. 2023). Activations in the visual cortex may reflect mechanisms by which visual information is encoded and processed during fear extinction learning, potentially influenced by interactions with the cerebellum. Cerebello‐frontal connections are well known, particularly with the dlPFC, which plays a crucial role in attentional processes related to fear conditioning (Lissek et al. 2017; Middleton and Strick 2001). Stronger occipital activation suggests enhanced sensory engagement, possibly increasing attention to CS‐related visual cues, and hereby improving extinction learning and/or consolidation.

Stimulation may also have enhanced attention to visual contextual cues—either by increasing attention to the CS within its context, promoting context processing or facilitating CS‐context memory consolidation. In our study design context remained constant during acquisition training, extinction training and recall, which rules out renewal effects. However, stronger binding to the extinction context could have reduced spontaneous recovery, i.e., the return of the initial fear association over time. One cannot exclude, however, that direct occipital stimulation effects have at least partially contributed. ctACS may have affected 4–8 Hz oscillations in visual cortical areas, which are associated with the processing of visual stimuli (Gao et al. 2021; Levy et al. 2017; Tang et al. 2023).

During recall, the main differences in the fMRI signal between the verum and sham groups were observed at the time when the US was presented in reinforced acquisition trials but did not occur in the recall trials (contrast ‘no‐US post CS+ > no‐US post CS‐‘). Since spontaneous recovery was observed in the sham group, the US was expected during the initial recall trials. The US omission in early recall was therefore unexpected in the sham but not the verum group. fMRI signal was significantly higher in the precentral gyrus (M1) in the sham group. M1 is known to be activated during fear acquisition training (Andreatta et al. 2015), which was also the case in the present study (Figure 5A, Table S4). In parametric modulation, that is activation related to model‐based prediction errors at the time of the US (which did not occur), significantly higher activations were found in the sham group in frontal cortex, including ACC, and parietal cortex. Finally, volume of interest analyses showed significantly higher beta values towards the CS+ compared to the CS‐ in the sham group, but not in the verum group in the right insula related to the prediction of the US in recall and its unexpected omission in early recall. A similar pattern in early recall was found in left insula and bilateral ACC, and a lesser extent in the cerebellum, but differential responses to the CS+ and CS‐ were not significantly different between groups.

The dominance of the right insula aligns with the well‐established right‐hemispheric lateralization of negative emotional control (Güntürkün et al. 2020). It is worth noting that ctACS was applied to the right cerebellum. While cerebellar projections are strongest to the contralateral cortex, ipsilateral connections are also present (Sultan et al. 2012).

Insular cortex and ACC, but also the cerebellum, are part of the neural network involved in the acquisition of conditioned fear responses (Buchel et al. 1999; Buchel et al. 1998; Doubliez et al. 2023; Fullana et al. 2016). A meta‐analysis by Fullana et al. (2018) found these regions—rather than areas typically linked to extinction learning, such as the vmPFC, hippocampus, and amygdala—to be most consistently activated during extinction training. The authors concluded that the insula and ACC activations, but also the cerebellum, during extinction and recall may reflect persistent fear acquisition‐related activity. Our findings are consistent with this interpretation. However, we observed higher activations in the sham compared to the verum group only in recall, and mainly at the time the US was expected but did not occur (which has not been analyzed by Fullana et al. 2018, who focused on the contrast ‘CS+ > CS‐’).

Applying 6 Hz ctACS during extinction training may prevent spontaneous recovery by promoting the downregulation of the initial fear memory. This interpretation is supported by recent findings in rodents. Frontera et al. (2023) found that output of the fastigial nuclei (FN) reduces theta oscillations in the rodent equivalent of the ACC (i.e., the prelimbic cortex within the dorsomedial prefrontal cortex, dmPFC), which is a necessary requirement of extinction learning. Notably, they also showed that FN in mice projects via the thalamus preferentially to the dmPFC rather than the vmPFC, which is commonly associated with extinction learning (Dunsmoor et al. 2019; Milad et al. 2007). Cerebellar granule (and Golgi) cells have been shown to oscillate in the theta frequency range (Dugue et al. 2009; Hoffmann and Berry 2009), suggesting that theta ctACS may enhance their recruitment. How this affects oscillations in cerebral areas is hard to predict, given that granule cells are connected to the (inhibitory) Purkinje cells, which project onto cerebellar nuclei that inform cortical areas via the thalamus (Popa et al. 2019). EEG recordings could help determine whether ctACS modulates theta oscillations in the prefrontal cortex and in which direction (increase or decrease). This will be an important area of interest for future research.

The present findings may have clinical implications. Extinction learning forms the basis of exposure therapy, a widely used technique for addressing fear memories associated with conditions such as posttraumatic stress disorder (PTSD; Milad et al. 2008). Our findings suggest that 6 Hz ctACS may be a potential way to enhance the efficacy of exposure therapy. Most studies aiming to enhance extinction have administered transcranial direct current stimulation (tDCS) directed to cerebral cortical areas, particularly parts of the prefrontal cortex. Findings, however, are heterogeneous, which is a common observation in many tDCS studies (Adams et al. 2020; Markovic et al. 2021, for review). tDCS is direction‐dependent, which makes it difficult to predict the outcome, particularly in highly convoluted structures like the cerebellum (Oldrati and Schutter 2018). tACS uses an oscillating current that can entrain neural oscillations at specific frequencies and is suggested to be largely direction‐independent. Thus, cerebellar tACS may reduce variability in outcomes and offer better reproducibility compared to cerebellar tDCS. Prior to proceeding to studies in patients, however, further studies are needed to validate the present findings, including testing different timings of application and stimulation frequencies. Furthermore, findings need to be replicated using a three‐day design, with fear acquisition and extinction training being performed on separate days. Of note, in a previous study of our group, we did not see ctACS effects on fear extinction learning (Schellen et al. 2024). The paradigm used, however, was different, including changes of context, and there were flaws in the design (reinforcement of the CS‐ which was not intended).

Limitations of the present study are inconsistencies in the fMRI data across phases and contrasts applied. For example, one would have also expected higher fMRI signal in the sham group in fear‐related areas such as the insular cortex and ACC related to the prediction of the US (contrast ‘CS+ > CS‐‘) during extinction training and recall, which was not the case. Likewise, parametric modulation at the time of US omission showed group differences in the ACC, but not the insular cortex, with the right insula showing the most prominent group differences in the VOI analysis. Effect size analysis showed that in some contrasts, significance may not have been reached because the variance was too high, the clusters too small, or the sample size too low. Thus, the findings are largely preliminary, and to provide definitive mechanistic insights, they need to be confirmed in future studies.

In sum, the present data suggest that theta cerebellar tACS enhances recall of fear extinction memory possibly by decreasing activation within the fear conditioning network. Enhanced attention to the conditioned stimulus or to visual contextual cues during extinction may have contributed to these effects.

## Author Contributions

**Giorgi Batsikadze:** investigation, conceptualization, methodology, software, formal analysis, visualization, project administration, supervision, writing – original draft, writing – review and editing. **Zsofia Spisak:** formal analysis, software, writing – review and editing. **Philippe Zeidan:** investigation. **Michael Klein:** investigation, formal analysis. **Enzo Nio:** formal analysis, software. **Thomas M. Ernst:** methodology, software, validation, resources, data curation, writing – review and editing. **Nicolas Diekmann:** methodology, formal analysis, software, writing – review and editing. **Sophia Göricke:** resources. **Sen Cheng:** conceptualization, methodology, writing – review and editing. **Christian J. Merz:** conceptualization, methodology, writing – review and editing. **Fatemeh Yavari:** conceptualization, methodology, software, writing – review and editing. **Michael A. Nitsche:** conceptualization, methodology, writing – review and editing. **Andreas Thieme:** investigation, formal analysis, writing – original draft, writing – review and editing. **Dagmar Timmann:** conceptualization, methodology, supervision, project administration, funding acquisition, writing – original draft, writing – review and editing.

## Funding

This work was supported by a grant from the German Research Foundation (DFG; project number 316803389—SFB 1280) to D.T. (subproject A05), C.J.M. (subproject A09), M.N. (subproject A06) and S.C. (subproject F01). At the time of this study A.T. held a position that was funded in part by the University Medicine Essen Clinician Scientist Academy (UMEA) and the German Research Foundation (DFG; grant no. FU356/12‐2).

## Ethics Statement

The study was approved by the Ethics Committee of the University Hospital Essen (proposal ID 16–7255‐BO).

## Conflicts of Interest

The authors declare no conflicts of interest.

## Supporting information


**Figure S1:** US/no‐US related skin conductance response (SCR) amplitudes and corresponding relative treatment effect (RTE) estimates during habituation, fear acquisition training, extinction training and recall.
**Figure S2:** (A, B) Log‐transformed mean skin conductance response (SCR) amplitudes for individual trials for habituation, acquisition training, extinction training and recall phases measured in (A) conditioned stimulus (CS) and (B) unconditioned response (US/no‐US) window. (C) Mean log‐transformed differential SCRs (SCR to CS+ minus SCR to CS‐; SCR_diff_) for individual trials for habituation, acquisition training, extinction training and recall phases, measured in CS window.
**Figure S3:** Median ratings regarding (A) valence, (B) arousal, (C) fear and (D) US expectancy obtained using a Likert‐scale ranging from 1 (*“very pleasant”/“very calm”/“not afraid”*, “US not expected”, respectively) to 9 (*“very unpleasant”/“very nervous”/“very afraid”, “US expected”*, respectively). The median values are represented by horizontal lines, and the whiskers extend from the first to the third quartile. The sham group is represented by red colors, the verum group by blue colors. Dark colors: CS+, light colors: CS‐.
**Figure S4:** Relative treatment effect (RTE) estimates for mean *β* values related to prediction of the aversive stimulus, extracted from six volumes of interest (VOIs). (A, B) cerebellum (lobule VI and Crus I); (C, D) insular cortex; and (E, F) anterior cingulate cortex (ACC) on day 1 (fear acquisition and extinction training) and day 2 (recall) shown across blocks. Panels A, C, and E display RTE estimates for the left hemisphere; panels B, D, and F show values from the right hemisphere.
**Figure S5:** Relative treatment effect (RTE) estimates for mean *β* values related to the omission of the aversive stimulus, extracted from six volumes of interest (VOIs). (A, B) cerebellum (lobule VI and Crus I); (C, D) insular cortex; and (E, F) anterior cingulate cortex (ACC) on day 1 (fear acquisition and extinction training) and day 2 (recall) shown across blocks. Panels A, C, and E display RTE estimates for the left hemisphere; panels B, D, and F show values from the right hemisphere.
**Table S1:** Results of the non‐parametric ANOVA‐type statistics (ATS) for repeated measures for skin conductance responses (SCRs), differential skin conductance responses (SCR_diff_), valence, arousal, fear, US expectancy and stimulation side‐effect ratings comparing verum and sham groups.
**Table S2:** Results of side‐effects questionnaires—Self‐reported median ratings and interquartile range (in brackets) prior and post ctACS administration.
**Table S3:** Results of the non‐parametric ANOVA‐type statistics (ATS) for repeated measures for individual trials of skin conductance responses (SCRs) and differential skin conductance responses (SCR_diff_).
**Table S4:** Fear acquisition training. Activation clusters are reported. Listed below are all local maxima within significant clusters (*p* < 0.05, corrected for multiple comparisons using a cluster‐forming threshold of *Z* = 3.1) and respective effect sizes (Cohen's *d*) with approximate 95% confidence intervals. The anatomical locations of these maxima were determined by referencing the Harvard‐Oxford Cortical and Subcortical, and Cerebellar Atlases in MNI152 space following normalization with FLIRT. Only structures with a probability of 25% or higher are reported. For regions where the Harvard‐Oxford atlas was insufficient for identification, manual identification was conducted. Brain areas manually identified using the Multi‐Contrast Anatomical Subcortical Structures (MASSP) atlas (Bazin et al. 2020) are indicated in square brackets. Percentages represent the likelihood of anatomical localization.
**Table S5:** Fear extinction training and recall. Activation clusters are reported. Listed below are all local maxima within significant clusters (*p* < 0.05, corrected for multiple comparisons using a cluster‐forming threshold of *Z* = 3.1) and respective effect sizes (Cohen's *d*) with approximate 95% confidence intervals. The anatomical locations of these maxima were determined by referencing the Harvard‐Oxford Cortical and Subcortical, and Cerebellar Atlases in MNI152 space following normalization with FLIRT. Only structures with a probability of 25% or higher are reported. For regions where the Harvard‐Oxford atlas was insufficient for identification, manual identification was conducted. Brain areas manually identified using the Multi‐Contrast Anatomical Subcortical Structures (MASSP) atlas (Bazin et al. 2020) are indicated in square brackets. Percentages represent the likelihood of anatomical localization.
**Table S6:** Fear acquisition training. Parametric modulation of learning model‐derived prediction parameters. Activation clusters are reported. Listed below are all local maxima within significant clusters (*p* < 0.05, corrected for multiple comparisons using a cluster‐forming threshold of *Z* = 3.1) and respective effect sizes (Cohen's *d*) with approximate 95% confidence intervals. The anatomical locations of these maxima were determined by referencing the Harvard‐Oxford Cortical and Subcortical, and Cerebellar Atlases in MNI152 space following normalization with FLIRT. Only structures with a probability of 25% or higher are reported. For regions where the Harvard‐Oxford atlas was insufficient for identification, manual identification was conducted. Brain areas manually identified using the Multi‐Contrast Anatomical Subcortical Structures (MASSP) atlas (Bazin et al. 2020) are indicated in square brackets. Percentages represent the likelihood of anatomical localization.
**Table S7:** Fear extinction training and recall. Parametric modulation of learning model‐derived prediction parameters. Activation clusters are reported. Listed below are all local maxima within significant clusters (*p* < 0.05, corrected for multiple comparisons using a cluster‐forming threshold of *Z* = 3.1) and respective effect sizes (Cohen's *d*) with approximate 95% confidence intervals. The anatomical locations of these maxima were determined by referencing the Harvard‐Oxford Cortical and Subcortical, and Cerebellar Atlases in MNI152 space following normalization with FLIRT. Only structures with a probability of 25% or higher are reported. For regions where the Harvard‐Oxford atlas was insufficient for identification, manual identification was conducted. Brain areas manually identified using the Multi‐Contrast Anatomical Subcortical Structures (MASSP) atlas (Bazin et al. 2020) are indicated in square brackets. Percentages represent the likelihood of anatomical localization.
**Table S8:** Results of the non‐parametric ANOVA‐type statistics (ATS) for repeated measures on mean *β* values comparing cerebellar verum and sham groups.

## Data Availability

The data that support the findings of this study are available from the corresponding author upon reasonable request.
